# Epidemiology of Signet Ring Cell Adenocarcinomas

**DOI:** 10.3390/cancers12061544

**Published:** 2020-06-11

**Authors:** Matthew G.K. Benesch, Alexander Mathieson

**Affiliations:** Discipline of Surgery, Faculty of Medicine, Memorial University of Newfoundland, St. John’s, NL AlB 3V6, Canada; alex.mathieson@med.mun.ca

**Keywords:** diffuse type, *CDH1*, E-cadherin, histopathology, chemotherapy, radiotherapy, surgery, cancer

## Abstract

Signet ring cell adenocarcinomas (SRCCs) are a rare histological subtype of adenocarcinomas with a poor prognosis, typically due to advanced disease at diagnosis. A signet ring cell, mimicking its moniker, contains abundant intracytoplasmic mucin that pushes the nucleus to the periphery. In these cancers, this cell feature comprises more than 50% of the tumor. Despite predilection for the gastrointestinal tract, and in particular the stomach, primary SRCCs are also described in other sites, typically in case reports. This literature, however, lacks a standardized overview of the SRCC disease entity. Using a retrospective cohort approach, we summarize the clinicodemographic and mortality outcomes of SRCCs in thirteen primary sites, comprising 95% of all SRCCs in the Surveillance, Epidemiology, and End Results Program (SEER), a population-level cancer database covering nearly one-third of the United States population. SRCCs general trends compared to matching nonvariant adenocarcinomas are earlier age of onset, with initial presentation favoring higher rates of regional or distant disease presentation and poor tumor differentiation. After multivariable analysis, SRCCs typically have worse overall survivals, but substantial variances exist depending on tumor location. Identifying SRCCs at earlier disease stages is likely the single most important intervention to improving outcomes for these patients.

## 1. Introduction—Overview of Signet Ring Cell Adenocarcinomas

**Figure 1:**
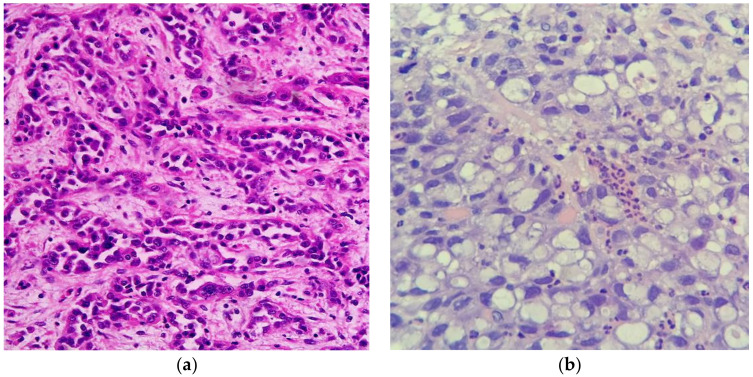
(**a**) Representative histological slide of poorly differentiated conventional gastric adenocarcinoma. (**b**) Representative histological slide of gastric signet adenocarcinoma, illustrating mucin-filled cytoplasm with nucleus pushed to the periphery. Figures sourced from Wikimedia Commons, public domain [6,7].

Signet ring cell adenocarcinomas (SRCCs) have been described in the literature since the 1950s, with most initial papers describing these cancers in the colon and urinary bladder [1,2]. Histologically, these cancers have the appearance of a signet ring. A signet ring has a flattened surface at the top of the ring with an engraved or raised symbol, historically used to stamp a seal into correspondence. Comparatively, these cells acquire a signet cell morphology due to accumulation of abundant mucin in cytoplasm, leading to nuclei dislocation to the periphery [3] (Figure 1). Formally, a cancer is labelled a SRCC if greater than 50% of tumor cells show prominent intracytoplasmic mucin and an eccentrically placed crescent-shaped nucleus [4], whereas adenocarcinomas with less than 50% signet ring cells are classified as “adenocarcinomas with a signet ring cell component” [5]. SRCCs are distinct from mucinous adenocarcinomas in that in the latter greater than 50% of the tumor consists of extracellular mucin [5].

The systematic study of these cancers is limited due to the rarity of this histological diagnosis. For gastric cancers, the World Health Organization (WHO) classification lists SRCCs under the category of diffuse or poorly cohesive carcinomas, which is divided into SRCCs and other poorly cohesive carcinomas [5,8]. For colorectal cancers, SRCCs are a recognized histological subtype of adenocarcinomas if they meet the definition as described previously [2]. For other cancer sites, SRCCs are extremely rare (typically less than 0.5% of diagnoses). Hence for these sites, SRCCs are described as patterns rather than subtypes as there is limited evidence as to the clinical relevance [5]. Nevertheless, cancers with SRCC morphology are recognized by the International Classification of Diseases for Oncology (ICD)-0-3 classification as a unique entry (8490/3) under the larger umbrella of Cystic, Mucinous and Serous Neoplasms [9].

The ability to characterize rare cancers requires collective input of small numbers of cases from individual institutions into registries that cover large populations. With sufficient subsequent analytical power, this enables researchers to make meaningful conclusions beyond that provided in case reports or case series studies. Most of our knowledge about the demographics and prognosis of SRCCs comes from the summaries of population-level registries of gastric and colon cancers [10,11], with supplementation from case series on SRCCs from other sites. However, there is no study that systematically and robustly compares SRCCs by site to matching nonvariant cases.

The Surveillance, Epidemiology, and End Results (SEER) database is a population-based cancer registry managed by the National Cancer Institute, currently encompassing about one-third of the United States with near universal capture of cases as regional registries were added to the program since 1975 [12]. It has become an invaluable resource for enumerating histopathological data with survival and mortality data across all cancer sites, demographics, and time [13]. In this retrospective site-stratified analysis, we overcome the limitations imposed by the overall low incidence of SRCCs across tumor sites by investigating their clinicopathological characteristics and survival outcomes comparable to all other cancers by site, with subgroup analysis to major nonvariant types using the SEER database. These findings will provide a more through epidemiological description and quantification of this poorly characterized rare cancer entity with a historically poor prognosis.

## 2. Analysis of Signet Ring Cell Adenocarcinomas by Site

**Figure 2:**
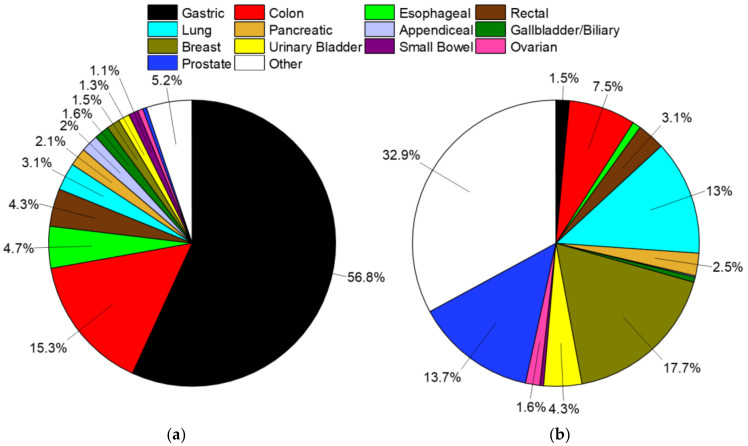
(**a**) Distribution of signet ring cell tumors in SEER, 1975–2016, total of 41,847 cases. (**b**) Distribution of all solid (non-blood borne), non-signet ring cell tumors in SEER, 1975–2016, total of 9.56 million cases. In both plots data labels are percentages. Markers omitted if less than 1%.

Subsections are presented in order of the percentage of SRCC cases relative to all cases of SRCC (Figure 2). First presented within each subsection is a demographics table with all included cases of cancers for that site, followed by the most common nonvariant histological type(s), and then SRCC cases. The second table presents both univariate and multivariable analyses for cause-specific mortality according the same demographic, histopathological, and treatment variables as the first table. Here, we present the hazard ratios for signet ring cell cancers compared to all other non-signet ring cell cancers within the site of interest. We then provide a sub-analysis of nonvariant histological type(s) to signet ring cell cancers to provide a representative comparator of risk to common cancer types within each site.

**Figure 3:**
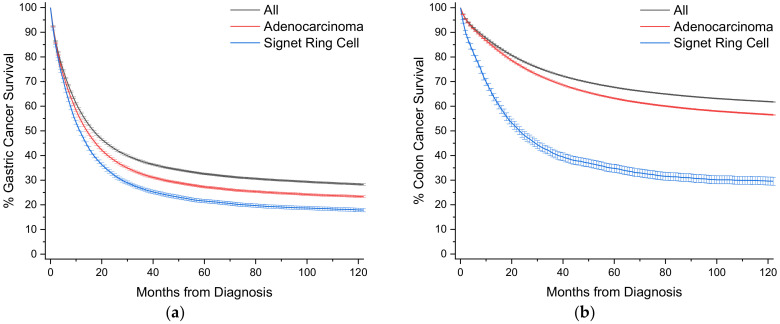

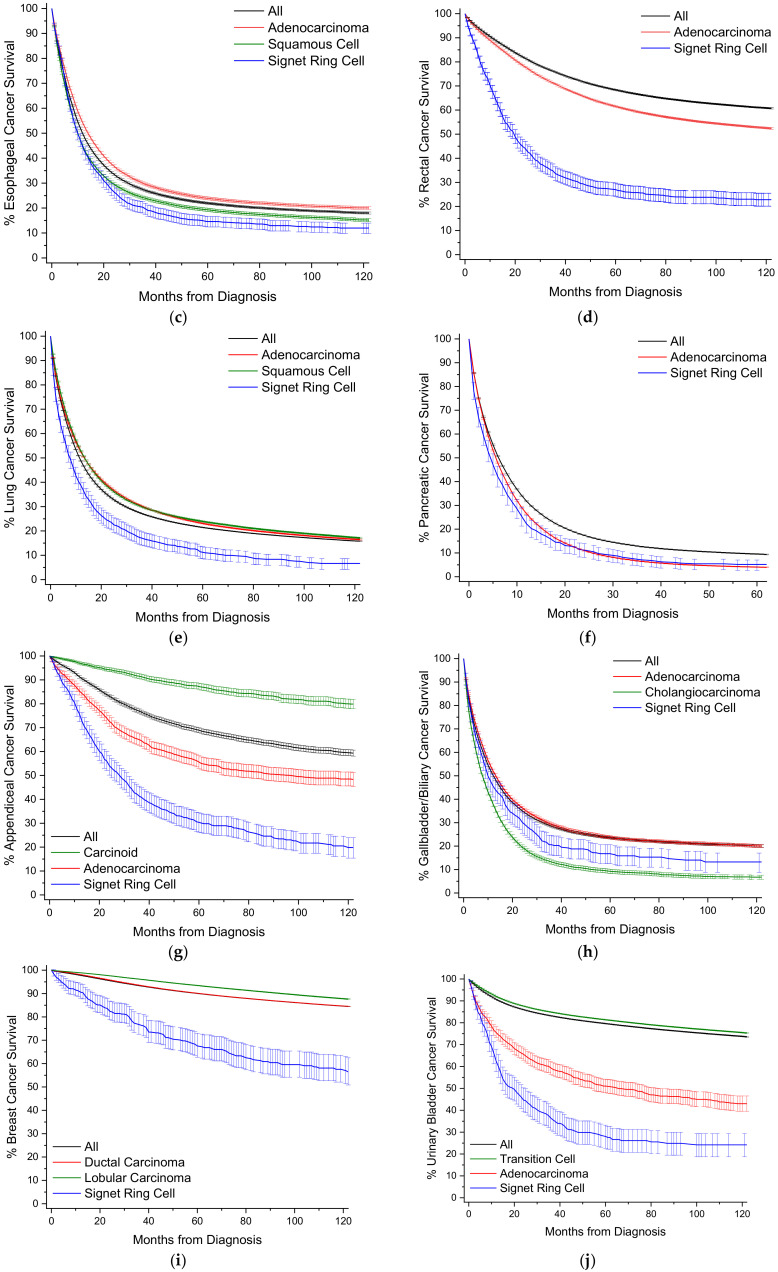

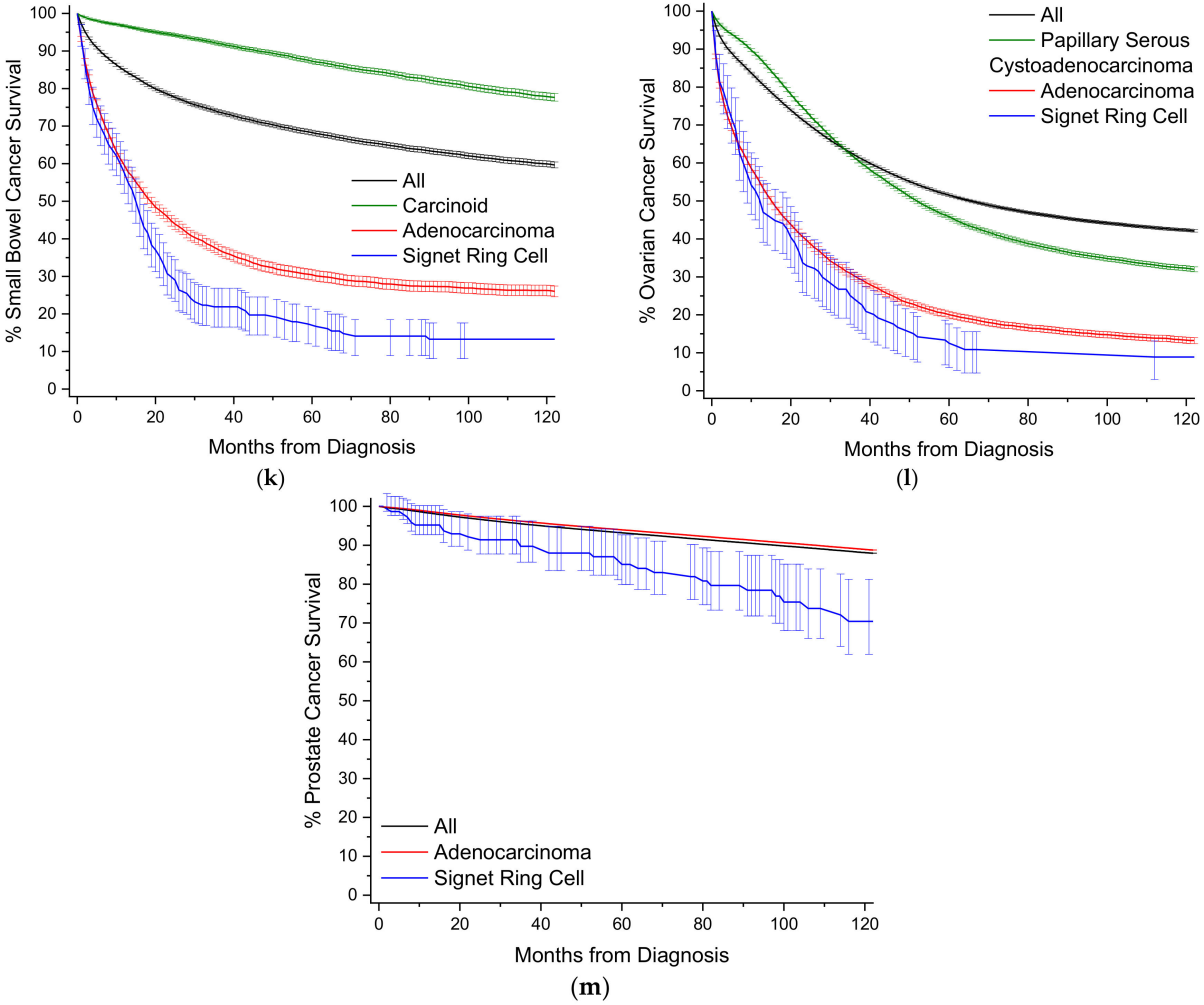
Kaplan-Meier survival curves. All survivor functions are shown with 95% confidence intervals. (**a**) Gastric cancer. (**b**) Colon cancer. (**c**) Esophageal cancer. (**d**) Rectal cancer. (**e**) Lung cancer. (**f**) Pancreatic cancer. (**g**) Appendiceal cancer. (**h**) Gallbladder/Biliary cancer. (**i**) Breast cancer. (**j**) Urinary Bladder cancer. (**k**) Small Bowel cancer. (**l**) Ovarian cancer. (**m**) Prostate cancer. In these curves, “All” represents the curves for all cancers within that site, with subtypes shown as labelled.

To provide a visual overview across all cancer sites, Kaplan-Meier survival curves with 95% confidence intervals are also presented (Figure 3).

### 2.1. Gastric

SRCCs comprise 16.8% of all cases of gastric cancer, and nearly 57% of all SRCC cases (Table 1, Figure 2). When compared to nonvariant adenocarcinomas, SRCCs have an earlier mean age of onset of 6.6 years. The proportion of female cases increases from 34.4% to 48.0% for SRCCs compared to adenocarcinomas where two-thirds of cases occur in males. These cancers are most often detected at a distant or metastatic stage (43.0% vs. 37.3%) with a poor tumor grade (75.4% vs. 52.1%). Surgical and radiotherapy trends are similar in both groups, while SRCC patients are more often treated with chemotherapy (49.5% vs. 41.4%). Survival is significantly worse at all time points for SRCC patients, with 5- and 10-year survival at 19.2% vs. 25.8% and 16.0% vs. 22.1%, respectively (Figure 3a, Table 1). Median survival time is 10.2 months compared to 13.5 months for adenocarcinomas. When compared to all other gastric cancers, SRCCs have a hazard ratio (HR) of 1.23 (95% CI: 1.21–1.26), and 1.11 (95% CI: 1.08–1.13) to adenocarcinomas, after multivariate analyses (Table 2). 

### 2.2. Colon

Colon cancers are analyzed separately from cancers arising from the appendix and rectum (Section 2.4 and Section 2.7). Carcinoids rather than nonvariant adenocarcinomas are the most common cancer subtype in the appendix, and rectal cancer is heavily treated with radiotherapy, unlike colon cancers. SRCCs comprise 1.0% of all cases of colon cancer, and 15.3% of all SRCC cases (Table 3, Figure 2). When compared to nonvariant adenocarcinomas, SRCCs have an earlier mean age of onset of 3.5 years, compared to 6.6 years for gastric SRCCs. Unlike gastric SRCCs, there is no gender bias towards females, but like gastric SRCCs, these cancers are most often detected at a distant or metastatic stage (40.6% vs. 23.8%) with a poor tumor grade (68.5% vs. 18.5%). SRCC patients receive chemotherapy more often (47.4% vs. 33.6%), although chemotherapy uptake appears low despite most cancers presenting at stages regional and greater. Colon SRCC patients have worse overall survival relative to comparative nonvariant cancers, with 5- and 10-year survival at 33.6% vs. 61.8% and 28.6% vs. 55.0%, respectively (Figure 3b, Table 3). Median survival time is only 21.6 months compared to more than 10 years for adenocarcinomas. When compared to all other colon cancers, SRCCs have a HR of 1.45 (95% CI: 1.40–1.51), and 1.47 (95% CI: 1.41–1.53) to adenocarcinomas, after multivariate analyses (Table 4).

### 2.3. Esophageal

SRCCs comprise 2.6% of all cases of esophageal cancer, and 4.7% of all SRCC cases (Table 5, Figure 2). When compared to nonvariant adenocarcinomas, there is no overt difference in age of onset, unlike gastric and colon SRCCs. There is also no gender bias, and SRCCs are more often detected regionally rather than distally, unlike the adenocarcinomas. However, consistent with the SRCCs the majority are of poor grade (77.8% compared to 42.1% for adenocarcinomas). 

For comparative purposes, squamous cell cancers are also included, which have worse 5 and 10-year CSS to adenocarcinomas (18.3%, 14.6% vs. 21.9%, 17.8%). Compared to squamous cell, SRCCs have statistically significant worse survival (SRCC 5- and 10-year survival 13.6% and 11.4%, respectively) (Figure 3c, Table 5). Median survival time is 9.8 months for SRCCs, compared to 9.7 months and 13.2 months for squamous cell and adenocarcinomas, respectively. SRCCs have a HR of 1.15 (95% CI: 1.08–1.22) compared to all other cancers, 1.16 (95% CI: 1.09–1.24) to adenocarcinomas, and 1.09 (1.02–1.16) to squamous cell, after multivariate analyses (Table 6).

### 2.4. Rectal

SRCCs comprise 0.7% of all cases of rectal cancer, and 4.3% of all SRCC cases (Table 7, Figure 2). When compared to nonvariant adenocarcinomas, SRCCs have an earlier mean age of onset of 6.6 years like gastric SRCCs, compared to 3.5 years for colon SRCCs. Unlike gastric SRCCs, there a larger gender bias towards males compared to adenocarcinomas (65.3% vs. 58.6%). This bias is not present in colon SRCCs (Table 3). Similar to gastric and colon SRCCs, these cancers are most often detected at a distant or metastatic stage (33.0% vs. 20.1%) with a poor tumor grade (66.3% vs. 13.2%). SRCC patients receive both radiotherapy (55.6% vs. 46.4%) and chemotherapy more often (68.6% vs. 56.6%). Rectal SRCC patients have the worst overall survival relative to comparative nonvariant cancers of all the SRCCs, with 5- and 10-year survival at 24.9% vs. 59.8% and 19.8% vs. 50.7%, respectively (Figure 3d, Table 7). 

Median survival time is 16.8 months compared to more than 10 years for adenocarcinomas. When compared to all rectal cancers, SRCCs have a HR of 2.14 (95% CI: 1.99–2.29), and 2.10 (95% CI: 1.95–2.25) to adenocarcinomas, after multivariate analyses (Table 8).

### 2.5. Lung

SRCCs comprise 0.1% of all cases of lung cancer, and 3.1% of all SRCC cases (Table 9, Figure 2). When compared to nonvariant adenocarcinomas, SRCCs have an earlier mean age of onset of 3.9 years and 5.9 years relative to nonvariant adenocarcinomas and squamous cells, respectively. There is a gender bias towards males compared to adenocarcinomas (56.3% vs. 50.4%). Like most gastrointestinal tract SRCCs, relative to adenocarcinomas, lung SRCCs mainly present with distant/metastatic disease (73.7% vs. 58.4%) and poor grade (39.5% vs. 29.1%). Compared to both adenocarcinomas and squamous cells, SRCCs have much worse 5-year (9.7% vs. 21.6% vs. 22.5%) and 10-year (4.9% vs. 15.1% vs. 16.2%) survivals (Figure 3e, Table 9). Median survival time is 5.7 months for SRCCs, compared to about 12.5 months for both squamous cells and adenocarcinomas. SRCCs have a HR of 1.19 (95% CI: 1.10–1.28) compared to all other cancers, 1.24 (95% CI: 1.15–1.33) to adenocarcinomas, and 1.17 (1.09–1.26) to squamous cells, after multivariate analyses (Table 10).

### 2.6. Pancreatic

SRCCs comprise 0.4% of all cases of pancreatic cancer, and 2.1% of all SRCC cases (Table 11, Figure 2). The relative differences between SRCCs and nonvariant adenocarcinomas are not as large as in other cancer types, likely as pancreatic adenocarcinoma has among the worse survival outcomes of any cancer type. There is a slight gender bias towards males compared to adenocarcinomas (54.8% vs. 51.3%). Distant/metastatic disease and poor grade are the most common presentation for both SRCCs and adenocarcinomas. Treatment modalities are grossly similar in both groups. SRCC have slightly worse 1-year survival compared to adenocarcinomas (20.6% vs. 26.6%), but at 5-years, survival is slightly better, but not statistically significant (4.5% vs. 3.8%) (Table 11, Figure 3f). Median survival time is 3.5 months for SRCCs compared to 5.6 months for adenocarcinomas. When compared to all pancreatic cancers, SRCCs have a HR of 1.27 (95% CI: 1.16–1.40), and non-significantly 1.04 (95% CI: 0.95–1.14) to adenocarcinomas, after multivariate analyses (Table 12).

### 2.7. Appendiceal

SRCCs comprise 5.8% of all cases of appendiceal cancer, and 2.0% of all SRCC cases (Table 13, Figure 2). Data for carcinoid tumors is presented as this is the most common cancer subtype at 39.8% of cases. When compared to nonvariant adenocarcinomas, SRCCs have an earlier mean age of onset of 3.7 years. There is a large gender bias towards females compared to adenocarcinomas (62.5% vs. 47.1%), like as seen with gastric SRCCs. Typical among SRCCs, relative to adenocarcinomas, appendiceal SRCCs mainly present with distant/metastatic disease (62.2% vs. 29.9%) and poor grade (53.4% vs. 21.0%). SRCCs have worse 5-year (34.2% vs. 54.0%) and 10-year (24.1% vs. 47.2%) survivals relative to adenocarcinomas, with median survival times of 30 months and 82 months, respectively (Figure 3g, Table 9). Like colon and rectal cancers, SRCCs have a very high mortality HR of 3.50 (95% CI: 3.16–3.89) compared to all cancers, 8.29 (95% CI: 7.25–9.48) to carcinoids, and 2.00 (95% CI: 1.77–2.26) to adenocarcinomas, on univariate analysis. However, unlike colon and rectal cancers, after multivariable analysis, the HR of mortality for SRCCs compared to adenocarcinomas is equivalent (0.95 (95% CI: 0.82–1.09)) (Table 14).

### 2.8. Gallbladder/Biliary

SRCCs comprised 1.3% of all cases of gallbladder/biliary cancer, and 1.6% of all SRCC cases (Table 15, Figure 2). Similar to pancreatic cancers, the relative differences between SRCCs, nonvariant adenocarcinomas, and cholangiocarcinomas are not as large as in other cancer types, likely to their poor outcome. Mean age of onset is younger for SRCCs by about 4 years. The only clinically significant histopathological difference is that most SRCCs present with poor grade relative to adenocarcinomas and cholangiocarcinomas (71.6% vs. 25.9% vs. 12.2%). SRCC patients undergo surgery more often (70.1% vs. 60.8% vs. 20.3%). SRCC have slightly worse 5 year-survival compared to adenocarcinomas (16.2% vs. 22.1%) but better than cholangiocarcinomas (16.2% vs. 8.6%) (Figure 3h, Table 15). Median survival time is 10–12.5 months across the three subtypes. When compared to all gallbladder/biliary cancers, SRCCs have a HR of 1.12 (95% CI: 1.01–1.25), and non-significant HR of 1.10 (95% CI: 0.99–1.23) to adenocarcinomas, and 1.06 (0.94–1.20) to cholangiocarcinomas, after multivariate analyses (Table 16).

### 2.9. Breast

SRCC comprise 0.03% of all cases of breast cancer, and 1.5% of all SRCC cases (Table 17, Figure 2). When compared to ductal and lobular cancers, SRCC have an older age of onset by about 7 years, unique among the SRCCs. Nearly a third of SRCCs are detected at distant/metastatic stage, whereas less than 5% of ductal and lobular cancers present at this stage. These patients are somewhat less likely to receive surgery (71.9% vs. ~94–95% for ductal and lobular cancers), likely due to increase likelihood of metastatic presentations. SRCCs have much worse 5-year (65.1% vs. ~90%) and 10-year (55.1% vs. ~82%) survivals relative to both ductal and lobular cancers (Figure 3i, Table 17). SRCCs have a very high mortality HR of 3.63 (95% CI: 3.09–4.27) and 4.65 (95% CI: 3.96–5.47) compared to breast and lobular cancers, respectively, on univariate analysis. However, after multivariable analysis, the HR of mortality for SRCCs compared to ductal cancers is equivalent (1.16 (95% CI: 0.98–1.36)), and marginally statistically increased for lobular cancers (1.19 (95% CI 1.01–1.40)) (Table 18).

### 2.10. Urinary Bladder

SRCCs comprised 0.2% of all cases of urinary bladder cancer, and 1.3% of all SRCC cases (Table 19, Figure 2). Transition cell carcinomas comprise 94% of all urinary bladder cases, and adenocarcinomas 0.5% of all cases. Mean age of onset is younger for SRCCs by 5.6 years and 3.5 years compared to transition cell and adenocarcinomas, respectively. SRCCs primarily present with localized and regional disease, predominantly with poor grade. SRCC have much worse 5 year-survival compared to transition cell and adenocarcinomas (28.3% vs. 49.4% vs. 82.0%), respectively. (Figure 3j, Table 19). Median survival time is 15.6 months for SRCCs, and 57.6 months for adenocarcinomas. When compared to all other urinary cancers, SRCCs have a HR of 4.83 (95% CI: 1.01–1.25) on univariate analysis, 1.56 (95% CI: 1.11–1.54), after multivariate analyses (Table 20).

### 2.11. Small Bowel

SRCCs comprise 1.1% of all cases of small bowel cancer, and 1.3% of all SRCC cases (Table 21, Figure 2). Data for carcinoid tumors is presented as this is the most common cancer subtype at 53.4% of cases. When compared to nonvariant adenocarcinomas, SRCCs have an earlier mean age of onset of 3.7 years like appendiceal cancers, but a near identical age compared to carcinoids. There is no overt gender bias. Relative to adenocarcinomas, appendiceal SRCCs present with slightly increased rates of regional and distant disease (~80% vs. ~70%) and poor grade (70.0% vs. 30.8%). SRCCs have worse 5-year survival relative to adenocarcinomas and carcinoids (13.8% vs. 23.1% vs. 74.7%), respectively (Figure 3k, Table 21). Median survival time is 14 months for SRCCs and 16.2 months for adenocarcinomas. When compared to all small bowel cancers, SRCCs have a HR of 1.64 (95% CI: 1.43–1.88), 4.47 (95% CI: 3.57–5.60) to carcinoids, and 1.23 (1.06–1.41) to adenocarcinomas, after multivariate analyses (Table 22).

### 2.12. Ovarian

SRCCs comprise 0.2% of all cases of ovarian cancer, and 0.6% of all SRCC cases (Table 23, Figure 2). Data for papillary serous cystoadenocarcinoma tumors is presented as this is the most common cancer subtype at 24.3% of cases. When compared to nonvariant adenocarcinomas, SRCCs have an earlier mean age of onset of 7 years like gastric cancers, but a later age of 1.7 years compared to the cystoadenocarcinomas. Consistent across all three subtypes, distant stage is the most common presentation. SRCCs have a comparable 5-year survival relative to adenocarcinomas, and much worse compared to cystoadenocarcinomas (10.8% vs. 19.2% vs. 38.2%), respectively (Figure 3l, Table 22). Median survival time is 7.9 months for SRCCs and 12.5 months for adenocarcinomas. When compared to all other ovarian cancers, SRCCs have a HR of 1.56 (95% CI: 1.31–1.86), 1.87 (95% CI: 1.56–2.23) to cystoadenocarcinomas, and 1.30 (1.09–1.56) to adenocarcinomas, after multivariate analyses (Table 24).

### 2.13. Prostate

SRCCs comprise 0.02% of all cases of prostate, and 0.4% of all SRCC cases (Table 25, Figure 2). Prostate cancer is predominantly nonvariant adenocarcinomas at 96.0% of all cases. When compared to nonvariant adenocarcinomas, SRCCs have an earlier mean age of onset of 1.7 years, but this is not statistically significant. SRCCs have a higher rate of detection at the distant/metastatic stage (13.2% vs. 4.2%), and most are poor grade differentiation (90.1% vs. 37.3%). There are no statistically significant differences among treatment modalities. SRCCs have worse 5-year survival relative to adenocarcinomas, (83.6% vs. 93.4%), respectively (Figure 3m, Table 25). When compared to all prostate cancers, SRCCs have a HR of 2.41 (95% CI: 1.71–3.39) and 2.63 (95% CI: 1.87–3.70) to adenocarcinomas, after univariate analyses. However, there are no significant differences after multivariable analyses (Table 26).

## 3. Discussion

To date, there has been no comparative analysis of SRCCs to site-matching nonvariant adenocarcinomas. Most of our knowledge about SRCCs is extrapolated from gastric cancers which comprise most SRCC cases. In this site, SRCCs are recognized as a distinct histological subtype, however, given the rarity of this disease entity in other sites, SRCCs are usually categorized instead as a pattern. This may have implications in patient management, as these patients and their families may benefit from more tailored screening or treatment regimens with the evolution of our understanding of SRCC tumor biology.

This analysis presents a quantification of the similarities among SRCCs across sites and points out some distinct differences. All sites have an earlier age of onset from 3–7 years for SRCCs, except for the breast and prostate cancer. Presentation at distant stages with poor differentiation are most common. Cancers of the large intestine have the worst survivals relative to nonvariant adenocarcinomas, whereas the mortality risk is not as largely increased for typically aggressive cancers sites (esophagus, pancreas, ovary). Even after adjusting for age, race, detection stage, grade, and treatment, the hazard risk for mortality relative to adenocarcinomas is most significant in the colon and rectum at 1.5–2.1. The risk for mortality remains significant after multivariate analysis in all sites except the pancreas, gallbladder, appendix, and prostate. This suggests that the primary tumor location may be an independent risk factor for cause-specific survival [14].

Conjectures regarding the inherit advanced disease pathology in SRCCs at presentation come from observations of the behavior of SRCCs in gastric cancer. The prototypical classification of gastric cancers is the Lauren classification from 1965 which divides them into intestinal and diffuse types [15]. As opposed to intestinal types, diffuse type cancers tend not to form macroscopic or fungating lesions. Diffuse type gastric cancers are defined by poorly-cohesive cells with no gland formation, and are predominately signet ring cell in nature [16]. This has evolved into the current dichotomy of diffuse gastric cancers: SRCCs and other poorly cohesive carcinomas. The two key pathogenic characteristics of SRCCs are their loss of cell-cell adhesion properties leading to their diffuse pattern of spread, and mucin accumulation [8]. It is not known if these two processes are connected.

In 1994, truncations in the protein E-cadherin, encoded by the gene *CDH1*, were first reported in diffuse gastric cancers [17]. Loss of E-cadherin expression is one on of the initiating steps in the epithelial-to-mesenchymal transition (EMT) process of metastasis, where loss of cell-cell contacts promotes a more plastic cellular cytoskeleton and anchorage-independent growth and survival during lymphatic and hematogenous spread of cells [18]. While this may be a mechanism behind gastric cancer SRCC aggressiveness, *CDH1* mutations are believed instead to contribute to earlier in tumor initiation [19]. In 1998, this mutation was confirmed to exist as an autosomal dominant germline gene mutation responsible for the hereditary diffuse gastric cancer (HDGC) syndrome [20,21]. These patients have to an 70% cumulative lifetime risk of gastric cancer with onset typically around age 40, and a 42% lifetime risk of lobular breast cancer [21]. Patients testing positive for *CDH1* mutations are recommended to undergo prophylactic gastrectomy by age 30 and high-risk breast cancer screening starting at age 35 [22,23]. The incidence of in situ signet ring cell lesions in prophylactic gastrectomy specimens is over 70% [24]. HDGC though comprises only 1–3% of all gastric cancers, whereas SRCCs comprise 10–18% of gastric cancers [24,25]. More widespread genetic testing is required to explore the genetic drivers of this entity. SRCCs is also the predominant histotype of linitis plastica, defined by thickened and rigid gastric walls caused by fibrous stroma reactions [26]. Linking *CDH1* mutations to SRCC sites is difficult given their rarity [16], though a case of appendiceal SRCC occurring with a gastric SRCC is reported [27].

The particularly aggressive nature of SRCCs and its earlier onset in colorectal cancers has been highlighted in the literature [28,29]. These cancers have higher frequency of *KRAS* and *BRAF* mutations compared with conventional cancers [30], correlating to shorter overall survival [31]. Colorectal SRCCs more readily metastasize to the peritoneum rather than the lung and liver like conventional adenocarcinomas [32]. SRCC in the rectosigmoid has been associated with a history of ulcerative colitis [33], and methylation of the *CDH1* promoter is seen in 93% of dysplastic biopsies compared to 6% of non-dysplastic biopsies in ulcerative colitis patients [34].

Several studies have looked at the effects of SRCC histology on therapeutic interventions. Neoadjuvant chemotherapy provided no survival benefit in patients with locally advanced gastric SRCC [35]. Esophageal SRCC predicts a poor response to preoperative chemoradiation, with no survival benefit from neoadjuvant treatment even if downstaging occurs [36]. This finding was further supported on systematic review [37]. SRCCs though with microsatellite instability-high (MSI-H tumors), are predominately in the right colon, and show good response to immune checkpoint therapy [38]. In prostate SRCCs, among the rarest SRCCs, with about 80 cases reported in the literature [39,40], a multimodal therapeutic approach is typically described, with hormonal treatment potentially adding longer-term benefit [41].

The presence of SRCCs outside of the gastrointestinal tract warrants careful immunohistochemical investigation to rule out metastasis, in addition to appropriate additional workup. This should include endoscopy with systematic random biopsies and narrow band imaging if gastric or colonic lesions are not seen macroscopically [42]. While estrogen receptor (ER) is most often positive in breast SRCCs, it can be negative in up to 20% of cases [43]. CK7 and CK20 expression patterns can often help in distinguishing from a gastric or colonic source [44]. The majority of ovarian SRCCs are metastatic from other primary lesions, typically of the gastrointestinal tract (Krukenberg tumors) [45]. In fact, the presence of SRCC histology carries a 98.4% positive predictive value for metastasis [46]. Consequently, any finding of ovarian SRCC warrants further investigation of an occult primary [47]. Predictors of a primary ovarian SRCC over a metastatic source include tumor unilaterality, younger age at diagnosis, and larger tumor size [46].

This study is not without limitations. This is a retrospective study and is therefore prone to selection bias. Our analysis is limited to binary treatment variables and does not take into consideration the temporal effects of advancements in therapeutic regimens over the course of the time period investigated. Stage and grade classifications have unique subtilities which are characteristic to the survival of individual cancer types, however, this study is focused on very broad definitions out of necessity. The rarity of SRCCs across most tumor sites does not allow for meaningful analysis outside of a population-level registry. Therefore, the primary strength of this work is our systematic analysis of one the largest cohorts of SRCC patients in a well-established population level cancer registry with rigorous quality improvement methodology [13].

## 4. Materials and Methods

### 4.1. Patient Selection

The National Cancer Institute’s SEER database was employed and included all amalgamated data from all 18 SEER cancer registries from 1975–2016, covering 28% of the United States population. Data release from the SEER database does not require informed patient consent. Permission to obtain the SEER database was obtained with the ID number 10095-Nov2018 via signed agreements [48].

The WHO formalized the definition of SRCCs as a histological subtype in gastric cancer in 1990 [8,25]. On the basis of jointpoint analysis (Jointpoint Trend Analysis Software, Version 4.8.0.1, Surveillance Research Program, National Cancer Institute, Calverton, MD, USA) completed on age-adjusted yearly incidence rates from 1975–2016, cases prior to 1992 were excluded assuming SRCCs were more likely to be diagnosed as adenocarcinomas prior to increased recognition of SRCCs as a distinct histological subtype in 1990 (Figure S1).

A complete outline of exclusion criteria and its effect on case numbers in presented in Table S1. A summary of the 18 SEER cancer registries and count of cancer cases within each registry is shown in Table S2. A complete definition of all variables and sites analyzed are presented in Tables S3 and S4.

### 4.2. Statistical Analysis

All data from the 18 SEER cancer registries was imported into Stata 15.1 (StataCorp LLC, College Station, TX, USA) for statistical analysis from SEER 1975–2016 Research Data in ASCII text format. A complete case analysis was completed after variable definition in Table S1. Baseline patient characteristics were compared with the *t* or *χ^2^* test as appropriate. Univariate and multivariable Cox proportional hazard regression was used to determine the association of mortality with cancer histology type, after adjusting for age, gender, race, detection stage, grade differentiation, surgery, radiotherapy, and chemotherapy. All hazard ratios are calculated with 95% confidence intervals. Use of surgery, radiotherapy, and chemotherapy as treatment modalities are taken as binary variables. All *p*-values are two-sided, and the threshold of 0.05 was used to determine significance. Survival curves were plotted using the Kaplan-Meier method. Graphs are produced using Origin Pro 2020 (OriginLab Corporation, Northampton, MA, USA). Incidence rates are calculated with SEER*Stat 8.3.6 (Surveillance Research Program, National Cancer Institute, Calverton, MD, USA), using SEER 18 (2000–2016 data) and are age-adjusted to the 2000 United Sates standard population with the age variable recode <1 year olds. Cause-specific survival and relative survival are both age standardized to the International Cancer Survival Standard 1—Age 15+ variable via the actuarial method (and Ederer II cumulative expected method for relative survival), using SEER*Stat 8.3.6 and SEER 18 (2000–2016 data).

## 5. Conclusions

This study aims to provide a standardized and systematic characterization of the broad demographical and histopathological features of SRCCs across all major sites and compare them to nonvariant histological types. This resource should provide insight into the outcome characteristics of this otherwise rarely quantified cancer subtype for researchers and clinicians. Where applicable, SRCC patients need to be stratified in clinical trials as effective therapeutic interventions are largely lacking for this population. Whereas histology has been instrumental in identification of SRCCs and epidemiology in characterization of their prognosis, their effective surveillance and treatment will depend on future omics breakthroughs that underpin understanding of their unique pathogenesis.

## Figures and Tables

**Table 1:** Baseline demographics and clinical characteristics by histology for gastric cancers.

Gastric	All	Adenocarcinoma	Signet Ring
**N (%)**	106,972 (100)	65,218 (61.0)	17,942 (16.8)
**Age (Years) (%)**			
0–14	38 (<0.1)	3 (<0.1)	4 (<0.1)
15–29	858 (0.8)	338 (0.5)	283 (1.6)
30–49	12,374 (11.6)	5688 (8.7)	3711 (20.7)
50–69	43,379 (40.6)	25,731 (39.5)	7847 (43.7)
70–85	39,088 (36.5)	25,902 (39.7)	4996 (27.8)
>85	11,235 (10.5)	7556 (11.6)	1101 (6.1)
**Mean (SD)**	67.0 (14.4)	68.6 (13.7)	61.9 (15.3)
**Gender (%)**			
Male	64,729 (60.5)	42,788 (65.6)	9334 (52.0)
Female	42,243 (39.5)	22,430 (34.4)	8608 (48.0)
**Race (%)**			
White	75,037 (70.1)	45,689 (70.1)	12,602 (70.2)
Black	14,627 (13.7)	8467 (13.0)	2199 (12.3)
Other	17,308 (16.2)	11,062 (17.0)	3141 (17.5)
**Detection Stage (%)**			
In Situ	1021 (1.0)	475 (0.7)	6 (<0.1)
Localized	27,653 (25.9)	14,922 (22.9)	3256 (18.1)
Regional	28,402 (26.6)	18,989 (29.1)	5586 (31.1)
Distant	38,065 (35.6)	24,333 (37.3)	7707 (43.0)
Unstaged	11,831 (11.1)	6499 (10.0)	1387 (7.7)
**Grade Differentiation (%)**			
Well	5762 (5.4)	2903 (4.5)	57 (0.3)
Moderate	21,817 (20.4)	18,465 (28.3)	405 (2.3)
Poor	52,910 (49.5)	34,003 (52.1)	13,531 (75.4)
Undifferentiated	2524 (2.4)	1015 (1.6)	486 (2.7)
Unknown	23,959 (22.4)	8832 (13.5)	3463 (19.3)
**Surgery (%)**			
Yes	56,058 (52.4)	33,371 (51.2) *	9088 (50.7) *
No	50,914 (47.6)	31,847 (48.8) *	8854 (49.3) *
**Radiotherapy (%)**			
Yes	21,836 (20.4)	15,520 (23.8)	3949 (22.0)
No	85,136 (79.6)	49,698 (76.2)	13,993 (78.0)
**Chemotherapy (%)**			
Yes	42,224 (39.5)	26,979 (41.4)	8896 (49.5)
No	64,748 (60.5)	38,239 (58.6)	9056 (50.5)
**Incidence Rate (95% CI)**			
All	7.57 (7.53–7.62)	4.50 (4.47–4.54)	1.24 (1.22–1.26)
Male	10.4 (10.3–10.5)	6.79 (6.72–6.86)	1.42 (1.39–1.45)
Female	5.37 (5.32–5.43)	2.71 (2.68–2.75)	1.11 (1.08–1.13)
**CSS % (95% CI)**			
1-year	55.7 (55.3–56.0)	52.9 (52.4–53.3)	45.8 (44.9–46.7)
2-year	41.7 (41.3–42.0)	37.1 (36.7–37.6)	30.0 (29.1–30.8)
5-year	31.1 (30.8–31.5)	25.8 (25.4–26.2)	19.2 (18.4–20.0)
10-year	27.0 (26.6–27.4)	22.1 (21.6–22.5)	16.0 (15.2–15.7)
Median (Months)	15.6	13.5	10.2
**RS %** **(95% CI)**			
1-year	53.7 (53.4–54.1)	51.0 (50.5–51.4)	44.3 (43.2–45.2)
2-year	39.8 (39.4–40.1)	35.3 (34.9–35.8)	28.4 (27.6–29.2)
5-year	29.1 (28.7–29.5)	24.0 (23.5–24.4)	17.9 (17.1–18.6)
10-year	24.1 (23.6–24.6)	19.3 (18.8–19.9)	14.2 (13.3–15.1)
Median (Months)	14.2	12.5	9.7

*p* < 0.05 for all comparisons between adenocarcinoma and signet ring cell comparisons, unless noted by * *p* ≥ 0.05. Incidence rates expressed per 100,000. CSS, cause-specific survival; RS, relative survival; CI, confidence interval.

**Table 2:** Derived univariate and multivariable Cox-proportional hazard ratios (HR) of mortality for gastric cancers.

Gastric	Signet Ring vs. Non-Signet Ring		Signet Ring vs. Adenocarcinoma	
HR (95% CI)	Univariate	Multivariable	Univariate	Multivariable
**Signet Ring Histology**	1.38 (1.36–1.41)	1.23 (1.21–1.26)	1.16 (1.14–1.19)	1.11 (1.08–1.13)
**Age (per 10 years)**	1.092 (1.085–1.098)	1.142 (1.134–1.149)	1.048 (1.041–1.054)	1.095 (1.087–1.102)
**Gender (Female)**	0.88 (0.87–0.90)	0.91 (0.89–0.92)	1.01 (0.99–1.03) *	0.96 (0.95–0.98)
**Race**				
Black	0.94 (0.92–0.96)	1.01 (0.99–1.04) *	0.99 (0.96–1.01) *	1.01 (0.99–1.04) *
Other	0.81 (0.79–0.83)	0.86 (0.84–0.88)	0.74 (0.72–0.76)	0.82 (0.80–0.84)
**Detection Stage**				
In Situ	0.40 (0.34–0.48)	0.38 (0.32–0.46)	0.36 (0.28–0.45)	0.29 (0.23–0.37)
Regional	2.79 (2.72–2.86)	3.01 (2.93–3.09)	2.13 (2.07–2.19)	2.63 (2.56–2.72)
Distant	7.13 (6.95–7.31)	5.62 (5.46–5.78)	5.75 (5.59–5.91)	4.95 (4.80–5.12)
Unstaged	3.68 (3.56–3.80)	2.23 (2.15–2.31)	3.69 (3.55–3.83)	2.17 (2.09–2.26)
**Grade Differentiation**				
Moderate	2.60 (2.46–2.75)	1.86 (1.76–1.97)	1.61 (1.51–1.71)	1.28 (1.20–1.36)
Poor	3.83 (3.64–4.04)	2.44 (2.31–2.57)	2.23 (2.11–2.36)	1.69 (1.59–1.79)
Undifferentiated	3.42 (3.19–3.68)	2.40 (2.23–2.58)	2.22 (2.04–2.41)	1.80 (1.66–1.96)
Unknown	2.47 (2.34–2.61)	1.57 (1.49–1.66)	2.55 (2.40–2.71)	1.49 (1.40–1.58)
**Surgery (Yes)**	0.316 (0.311–0.321)	0.409 (0.401–0.418)	0.315 (0.309–0.320)	0.417 (0.408–0.427)
**Radiotherapy (Yes)**	1.01 (0.99–1.03) *	1.02 (1.00–1.04) *	0.81 (0.80–0.83)	0.98 (0.95–1.00)
**Chemotherapy (Yes)**	1.26 (1.24–1.28)	0.66 (0.65–0.68)	1.05 (1.03–1.07)	0.63 (0.61–0.64)

*p* < 0.05 relative to reference unless noted by * *p* ≥ 0.05. Reference categories: Gender (Male), Race (White), Detection Stage (Localized), Grade differentiation (Well), Surgery (No), Radiotherapy (No), and Chemotherapy (No). CI, confidence interval.

**Table 3:** Baseline demographics and clinical characteristics by histology for colon cancers.

Colon	All	Adenocarcinoma	Signet Ring
**N**	448,221 (100)	283,919 (63.3)	4586 (1.0)
**Age (Years) (%)**			
0–14	32 (<0.1)	6 (<0.1)	10 (0.2)
15–29	1930 (0.4)	1066 (0.4)	140 (3.1)
30–49	38,386 (8.6)	24,725 (8.7)	649 (14.2)
50–69	182,891 (40.8)	113,023 (39.8)	1730 (37.7)
70–85	172,601 (38.5)	111,469 (39.3)	1582 (34.5)
>85	52,381 (11.7)	33,630 (11.8)	475 (10.4)
**Mean (SD)**	68.4 (13.6)	68.7 (13.6)	65.2 (16.4)
**Gender (%)**			
Male	220,451 (49.2)	138,746 (48.9) *	2276 (49.6) *
Female	227,770 (50.8)	145,173 (51.1) *	2310 (50.4) *
**Race (%)**			
White	356,033 (79.4)	224,658 (79.1)	3851 (84.0)
Black	55,559 (12.4)	34,885 (12.3)	429 (9.4)
Other	36,629 (8.2)	24,376 (8.6)	306 (6.7)
**Detection Stage (%)**			
In Situ	21,655 (4.8)	1975 (0.7)	1 (<0.1)
Localized	159,954 (35.7)	87,453 (30.8)	544 (11.9)
Regional	155,275 (34.6)	117,946 (41.5)	2078 (45.3)
Distant	93,274 (20.8)	67,485 (23.8)	1864 (40.6)
Unstaged	18,063 (4.0)	9060 (3.2)	99 (2.2)
**Grade Differentiation (%)**			
Well	38,192 (8.5)	18,437 (6.5)	25 (0.5)
Moderate	254,481 (56.8)	186,654 (65.7)	246 (5.4)
Poor	72,229 (16.1)	52,503 (18.5)	3143 (68.5)
Undifferentiated	7970 (1.8)	4397 (1.5)	433 (9.4)
Unknown	75,349 (16.8)	21,928 (7.7)	739 (16.1)
**Surgery (%)**			
Yes	394,923 (88.1)	251,754 (88.7)	3875 (84.5)
No	53,298 (11.9)	32,165 (11.3)	711 (15.5)
**Radiotherapy (%)**			
Yes	9237 (2.1)	6971 (2.5)	166 (3.6)
No	438,984 (97.9)	276,948 (97.5)	4420 (96.4)
**Chemotherapy (%)**			
Yes	125,723 (28.0)	95,441 (33.6)	2172 (47.4)
No	322,498 (72.0)	188,478 (66.4)	2414 (52.6)
**Incidence Rate (95% CI)**			
All	31.2 (31.1–31.3)	20.1 (20.0–20.2)	3.5 (3.4–3.6) **†**
Male	35.0 (34.9–35.2)	22.6 (22.5–22.7)	3.8 (3.7–4.0) **†**
Female	28.1 (28.0–28.3)	18.15 (18.06–18.24)	3.1 (3.0–3.3) **†**
**CSS % (95% CI)**			
1-year	83.5 (83.4–83.7)	83.3 (83.2–83.5)	64.0 (62.4–65.6)
2-year	75.9 (75.7–76.0)	74.8 (74.6–74.9)	47.5 (45.8–49.2)
5-year	64.6 (64.4–64.8)	61.8 (61.6–62.0)	33.6 (31.8–35.3)
10-year	58.5 (58.3–58.7)	55.0 (54.7–55.2)	28.6 (26.8–30.4)
Median (Months)	-	-	21.6
**RS %** **(95% CI)**			
1-year	82.1 (82.0–82.2)	81.9 (81.8–82.1)	61.4 (59.7–63.0)
2-year	74.8 (74.7–75.0)	73.8 (73.6–74.0)	45.4 (43.6–47.1)
5-year	64.2 (64.0–64.4)	61.7 (61.4–61.9)	31.7 (29.9–33.6)
10-year	57.8 (57.5–58.1)	54.7 (54.3–55.0)	26.3 (24.0–28.7)
Median (Months)	-	-	19.4

*p* < 0.05 for all comparisons between adenocarcinoma and signet ring cell comparisons, unless noted by * *p* ≥ 0.05. Incidence rates expressed per 100,000, except **†** (per 1 million). CSS, cause-specific survival; RS, relative survival; CI, confidence interval.

**Table 4:** Derived univariate and multivariable Cox-proportional hazard ratios (HR) of mortality for colon cancers.

Colon	Signet Ring vs. Non-Signet Ring		Signet Ring vs. Adenocarcinoma	
HR (95% CI)	Univariate	Multivariable	Univariate	Multivariable
**Signet Ring Histology**	2.69 (2.58–2.79)	1.45 (1.40–1.51)	2.31 (2.22–2.40)	1.47 (1.41–1.53)
**Age (per 10 years)**	1.149 (1.144–1.154)	1.242 (1.237–1.248)	1.089 (1.083–1.094)	1.212 (1.205–1.218)
**Gender (Female)**	1.03 (1.02–1.05)	0.96 (0.95–0.97)	0.98 (0.97–0.99)	0.96 (0.95–0.97)
**Race**				
Black	1.22 (1.20–1.24)	1.21 (1.19–1.23)	1.29 (1.26–1.31)	1.23 (1.21–1.25)
Other	0.91 (0.89–0.92)	0.93 (0.92–0.95)	0.92 (0.90–0.94)	0.94 (0.92–0.96)
**Detection Stage**				
In Situ	0.42 (0.39–0.44)	0.39 (0.36–0.41)	0.53 (0.45–0.62)	0.43 (0.37–0.51)
Regional	3.01 (2.96–3.07)	3.13 (3.08–3.19)	2.48 (2.43–2.53)	2.70 (2.64–2.76)
Distant	17.5 (17.2–17.8)	16.6 (16.3–17.0)	14.2 (13.9–14.5)	14.4 (14.1–14.7)
Unstaged	8.8 (8.5–9.0)	4.32 (4.19–4.45)	6.66 (6.42–6.91)	3.40 (3.27–3.54)
**Grade Differentiation**				
Moderate	1.73 (1.68–1.77)	1.25 (1.22–1.28)	1.37 (1.33–1.41)	1.17 (1.13–1.20)
Poor	3.18 (3.10–3.26)	1.80 (1.75–1.85)	2.34 (2.27–2.42)	1.65 (1.60–1.71)
Undifferentiated	3.19 (3.05–3.33)	1.98 (1.89–2.06)	2.37 (2.25–2.50)	1.73 (1.64–1.83)
Unknown	2.28 (2.22–2.35)	1.39 (1.35–1.43)	3.89 (3.75–4.03)	1.41 (1.36–1.46)
**Surgery (Yes)**	0.167 (0.165–0.170)	0.39 (0.38–0.40)	0.175 (0.172–0.178)	0.40 (0.39–0.41)
**Radiotherapy (Yes)**	2.14 (2.08–2.20)	1.08 (1.05–1.11)	1.83 (1.77–1.88)	1.08 (1.05–1.12)
**Chemotherapy (Yes)**	1.95 (1.93–1.98)	0.86 (0.85–0.87)	1.58 (1.56–1.60)	0.81 (0.80–0.82)

*p* < 0.05 relative to reference. Reference categories: Gender (Male), Race (White), Detection Stage (Localized), Grade differentiation (Well), Surgery (No), Radiotherapy (No), and Chemotherapy (No). CI, confidence interval.

**Table 5:** Baseline demographics and clinical characteristics by histology for esophageal cancers.

Esophageal	All	Adenocarcinoma	Squamous Cell	Signet Ring
**N**	59,989 (100)	30,860 (51.4)	20,634 (34.4)	1549 (2.6)
**Age (Years) (%)**				
0–14	3 (<0.1)	0 (0)	3 (<0.1)	0 (0)
15–29	109 (0.2)	78 (0.3)	11 (0.1)	5 (0.3)
30–49	4597 (7.7)	2583 (8.4)	1356 (6.6)	133 (8.6)
50–69	30,938 (51.6)	16,299 (52.8)	10,583 (51.3)	816 (52.7)
70–85	20,281 (33.8)	10,002 (32.4)	7333 (35.5)	521 (33.6)
>85	4061 (6.8)	1898 (6.2)	1348 (6.5)	74 (4.8)
**Mean (SD)**	66.5 (12.0)	65.9 (12.1)	67.0 (11.6)	65.8 (11.8)
**Gender (%)**				
Male	46,488 (77.5)	26,477 (85.8)	13,442 (65.1)	1346 (86.9)
Female	13,501 (22.5)	4383 (14.2)	7192 (34.9)	203 (13.1)
**Race (%)**				
White	49,399 (82.3)	29,242 (94.8)	12,696 (61.5)	1457 (94.1)
Black	7273 (12.1)	830 (2.7)	5767 (27.9)	45 (2.9)
Other	3317 (5.5)	788 (2.6)	2171 (10.5)	47 (3.0)
**Detection Stage (%)**				
In Situ	906 (1.5)	398 (1.3)	256 (1.2)	0 (0)
Localized	12,352 (20.6)	6673 (21.6)	4332 (21.0)	260 (16.8)
Regional	18,347 (30.6)	9326 (30.2)	6954 (33.7)	612 (39.5)
Distant	20,768 (34.6)	11,556 (37.4)	6170 (29.9)	542 (35.1)
Unstaged	7616 (12.7)	2907 (9.4)	2922 (14.2)	134 (8.7)
**Grade Differentiation (%)**				
Well	2710 (4.5)	1595 (5.2)	961 (4.7)	3 (0.2)
Moderate	18,923 (31.5)	10,368 (33.6)	7985 (38.7)	55 (3.6)
Poor	24,233 (40.4)	12,995 (42.1)	7505 (36.4)	1205 (77.8)
Undifferentiated	1068 (1.8)	392 (1.3)	185 (0.9)	41 (2.6)
Unknown	13,055 (21.8)	5510 (17.9)	3998 (19.4)	245 (15.8)
**Surgery (%)**				
Yes	18,433 (30.7)	11,160 (36.2)	4995 (24.2)	528 (34.1)
No	41,556 (69.3)	19,700 (63.8)	15,639 (75.8)	1021 (65.9)
**Radiotherapy (%)**				
Yes	32,376 (54.0)	15,984 (51.8)	12,892 (62.5)	914 (59.0)
No	27,613 (46.0)	14,876 (48.2)	7742 (37.5)	635 (41.0)
**Chemotherapy (%)**				
Yes	34,006 (56.7)	18,169 (58.9)	11,887 (57.6)	997 (64.4)
No	25,983 (43.3)	12,691 (41.1)	8747 (42.4)	552 (35.6)
**Incidence Rate (95% CI)**				
All	4.40 (4.37–4.44)	2.27 (2.24–2.29)	1.47 (1.45–1.49)	1.18 (1.13–1.24) †
Male	7.58 (7.51–7.65)	4.33 (4.28–4.38)	2.10 (2.06–2.13)	2.27 (2.15–2.39) †
Female	1.82 (1.80–1.85)	0.59 (0.58–0.61)	0.96 (0.93–0.98)	0.30 (0.26–0.34) †
**CSS % (95% CI)**				
1-year	48.2 (47.7–48.6)	52.7 (52.0–53.3)	43.8 (42.9–44.6)	43.7 (41.0–46.5)
2-year	31.5 (31.0–31.9)	34.8 (34.2–35.5)	28.0 (27.2–28.8)	25.5 (23.0–28.0)
5-year	20.0 (19.6–20.4)	21.9 (21.3–22.5)	18.3 (17.6–19.0)	13.6 (11.6–15.8)
10-year	16.1 (15.7–16.6)	17.8 (17.2–18.4)	14.6 (13.8–15.3)	11.4 (9.37–13.5)
Median (Months)	11.3	13.2	9.7	9.8
**RS % (95% CI)**				
1-year	46.1 (45.6–46.5)	50.6 (50.0–51.2)	41.6 (40.8–42.5)	42.6 (39.9–45.3)
2-year	29.5 (29.1–30.0)	33.0 (32.4–33.6)	25.9 (25.2–26.7)	24.2 (21.8–26.7)
5-year	17.8 (17.4–18.2)	20.0 (19.4–20.6)	15.8 (15.1–16.5)	12.4 (10.4–14.6)
10-year	13.1 (12.7–13.6)	15.0 (14.4–15.7)	11.0 (10.2–11.8)	10.0 (8.02–12.3)
Median (Months)	10.5	12.3	9.1	9.5

*p* < 0.05 for all comparisons among adenocarcinoma, squamous cell, and signet ring cell comparisons. Incidence rates expressed per 100,000, except **†** (per 1 million). CSS, cause-specific survival; RS, relative survival; CI, confidence interval.

**Table 6:** Derived univariate and multivariable Cox-proportional hazard ratios (HR) of mortality for esophageal cancers.

Esophageal	Signet Ring vs Non-Signet Ring		Signet Ring vs Adenocarcinoma		Signet Ring vs Squamous Cell	
HR (95% CI)	Univariate	Multivariable	Univariate	Multivariable	Univariate	Multivariable
**Signet Ring Histology**	1.19 (1.12–1.26)	1.15 (1.08–1.22)	1.30 (1.32–1.39)	1.16 (1.09–1.24)	1.06 (1.00–1.13) *	1.09 (1.02–1.16)
**Age (per 10 years)**	1.116 (1.106–1.126)	1.098 (1.088–1.108)	1.115 (1.102–1.129)	1.100 (1.086–1.114)	1.084 (1.069–1.100)	1.064 (1.048–1.080)
**Gender (Female)**	1.00 (0.97–1.02) *	0.93 (0.90–0.95)	1.07 (1.03–1.11)	0.99 (0.95–1.03) *	0.85 (0.82–0.88)	0.85 (0.83–0.88)
**Race**						
Black	1.30 (1.26–1.34)	1.21 (1.17–1.25)	1.20 (1.11–1.31)	1.07 (0.98–1.16) *	1.21 (1.17–1.26)	1.16 (1.11–1.20)
Other	1.08 (1.04–1.13)	1.00 (0.96–1.05) *	1.07 (0.98–1.16) *	0.92 (0.85–1.01) *	1.00 (0.95–1.06) *	0.97 (0.92–1.00) *
**Detection Stage**						
In Situ	0.29 (0.25–0.34)	0.27 (0.23–0.32)	0.29 (0.22–0.36)	0.27 (0.21–0.34)	0.35 (0.28–0.44)	0.28 (0.22–0.35)
Regional	1.59 (1.55–1.64)	1.85 (1.79–1.91)	1.91 (1.83–1.99)	2.18 (2.08–2.29)	1.23 (1.18–1.29)	1.43 (1.36–1.49)
Distant	3.46 (3.36–3.57)	3.25 (3.14–3.36)	4.52 (4.34–4.72)	3.96 (3.78–4.16)	2.36 (2.25–2.47)	2.36 (2.25–2.48)
Unstaged	2.30 (2.21–2.39)	1.76 (1.70–1.83)	2.91 (2.75–3.08)	2.02 (1.90–2.14)	1.64 (1.55–1.74)	1.39 (1.31–1.47)
**Grade Differentiation**						
Moderate	1.49 (1.41–1.57)	1.31 (1.24–1.39)	1.55 (1.43–1.66)	1.28 (1.19–1.38)	1.33 (1.22–1.45)	1.28 (1.18–1.40)
Poor	1.93 (1.83–2.04)	1.59 (1.51–1.68)	2.20 (2.05–2.37)	1.69 (1.57–1.82)	1.49 (1.36–1.62)	1.34 (1.23–1.47)
Undifferentiated	1.90 (1.74–2.07)	1.62 (1.48–1.77)	1.98 (1.74–2.26)	1.71 (1.50–1.95)	1.39 (1.17–1.65)	1.31 (1.10–1.56)
Unknown	1.40 (1.32–1.48)	1.18 (1.12–1.25)	1.40 (1.30–1.52)	1.18 (1.09–1.28)	1.28 (1.17–1.40)	1.19 (1.09–1.31)
**Surgery (Yes)**	0.39 (0.38–0.40)	0.49 (0.48–0.50)	0.32 (0.31–0.33)	0.44 (0.43–0.46)	0.55 (0.53–0.57)	0.58 (0.55–0.60)
**Radiotherapy (Yes)**	0.90 (0.88–0.92)	0.95 (0.92–0.97)	1.00 (0.97–1.02) *	1.02 (0.99–1.05) *	0.68 (0.66–0.71)	0.80 (0.77–0.84)
**Chemotherapy (Yes)**	0.87 (0.85–0.89)	0.59 (0.57–0.61)	1.04 (1.01–1.07)	0.59 (0.57–0.61)	0.63 (0.61–0.65)	0.57 (0.54–0.59)

*p* < 0.05 relative to reference unless noted by * *p* ≥ 0.05. Reference categories: Gender (Male), Race (White), Detection Stage (Localized), Grade differentiation (Well), Surgery (No), Radiotherapy (No), and Chemotherapy (No). CI, confidence interval.

**Table 7:** Baseline demographics and clinical characteristics by histology for rectal cancers.

Rectal	All	Adenocarcinoma	Signet Ring
**N**	194,109 (100)	119,273 (61.4)	1295 (0.7)
**Age (Years) (%)**			
0–14	10 (<0.1)	2 (<0.1)	1 (0.1)
15–29	1403 (0.7)	636 (0.5)	78 (6.0)
30–49	27,051 (13.9)	16,245 (13.6)	323 (24.9)
50–69	97,233 (50.1)	57,992 (48.6)	542 (41.9)
70–85	55,826 (28.8)	36,187 (30.3)	296 (22.9)
>85	12,586 (6.5)	8211 (6.9)	55 (4.2)
**Mean (SD)**	63.7 (13.7)	64.4 (13.6)	57.8 (16.9)
**Gender (%)**			
Male	110,203 (56.8)	69,952 (58.6)	845 (65.3)
Female	83,906 (43.2)	49,321 (41.4)	450 (34.7)
**Race (%)**			
White	154,869 (79.8)	96,639 (81.0)	1002 (77.4)
Black	19,448 (10.0)	10,550 (8.8)	130 (10.0)
Other	19,792 (10.2)	12,084 (10.1)	163 (12.6)
**Detection Stage (%)**			
In Situ	9251 (4.8)	730 (0.6)	3 (0.2)
Localized	78,378 (40.4)	37,686 (31.6)	156 (12.0)
Regional	64,405 (33.2)	50,775 (42.6)	643 (49.7)
Distant	30,903 (15.9)	23,972 (20.1)	427 (33.0)
Unstaged	11,172 (5.8)	6110 (5.1)	66 (5.1)
**Grade Differentiation (%)**			
Well	17,774 (9.2)	7599 (6.4)	12 (0.9)
Moderate	108,345 (55.8)	82,503 (69.2)	62 (4.8)
Poor	22,844 (11.8)	15,736 (13.2)	859 (66.3)
Undifferentiated	1999 (1.0)	934 (0.8)	90 (6.9)
Unknown	43,147 (22.2)	12,501 (10.5)	272 (21.0)
**Surgery (%)**			
Yes	156,833 (80.8)	94,549 (79.3)	901 (69.6)
No	37,276 (19.2)	24,724 (20.7)	394 (30.4)
**Radiotherapy (%)**			
Yes	71,720 (36.9)	55,224 (46.3)	720 (55.6)
No	122,389 (63.1)	64,049 (53.7)	575 (44.4)
**Chemotherapy (%)**			
Yes	86,677 (44.7)	67,533 (56.6)	888 (68.6)
No	107,432 (55.3)	51,720 (43.4)	407 (31.4)
**Incidence Rate (95% CI)**			
All	12.81 (12.75–12.87)	8.02 (7.97–8.06)	0.94 (0.89–0.99) †
Male	16.11 (16.01–16.21)	10.45 (10.37–10.53)	1.33 (1.24–1.42) †
Female	10.10 (10.03–10.17)	6.02 (5.96–6.07)	0.62 (0.57–0.68) †
**CSS % (95% CI)**			
1-year	86.4 (86.2–86.5)	85.3 (85.0–85.5)	61.9 (58.5–65.2)
2-year	78.4 (78.2–78.7)	76.0 (75.7–76.3)	41.2 (37.7–44.6)
5-year	64.8 (64.5–65.0)	59.8 (59.4–60.1)	24.9 (21.8–28.2)
10-year	56.8 (56.5–57.2)	50.7 (50.3–51.1)	19.8 (16.6–23.1)
Median (Months)	-	-	16.8
**RS % (95% CI)**			
1-year	85.2 (85.0–85.5)	84.1 (83.9–84.4)	59.8 (56.3–63.1)
2-year	77.6 (77.3–77.8)	75.1 (74.8–75.4)	39.4 (36.0–42.9)
5-year	64.5 (64.2–64.9)	59.5 (59.1–59.9)	23.2 (20.0–26.5)
10-year	56.5 (56.0–56.9)	50.2 (49.6–50.8)	18.5 (15.0–22.3)
Median (Months)	-	-	16.0

*p* < 0.05 for all comparisons among adenocarcinoma and signet ring cell comparisons. Incidence rates expressed per 100,000, except **†** (per 1 million). CSS, cause-specific survival; RS, relative survival; CI, confidence interval.

**Table 8:** Derived univariate and multivariable Cox-proportional hazard ratios (HR) of mortality for rectal cancers.

Rectal	Signet Ring vs. Non-Signet Ring		Signet Ring vs. Adenocarcinoma	
HR (95% CI)	Univariate	Multivariable	Univariate	Multivariable
**Signet Ring Histology**	3.42 (3.19–3.66)	2.14 (1.99–2.29)	2.70 (2.53–2.90)	2.10 (1.95–2.25)
**Age (per 10 years)**	1.242 (1.235–1.250)	1.310 (1.301–1.319)	1.178 (1.170–1.187)	1.245 (1.236–1.255)
**Gender (Female)**	0.94 (0.92–0.95)	0.93 (0.91–0.94)	0.97 (0.96–0.99)	0.95 (0.93–0.97)
**Race**				
Black	1.16 (1.13–1.19)	1.19 (1.16–1.22)	1.41 (1.36–1.45)	1.29 (1.25–1.32)
Other	0.88 (0.86–0.91)	0.93 (0.90–0.95)	0.96 (0.93–0.99)	0.97 (0.94–1.00) *
**Detection Stage**				
In Situ	0.37 (0.34–0.40)	0.38 (0.35–0.41)	0.46 (0.36–0.58)	0.35 (0.28–0.45)
Regional	2.80 (2.73–2.86)	2.92 (2.85–2.99)	2.00 (1.95–2.06)	2.35 (2.28–2.42)
Distant	14.6 (14.2–14.9)	12.6 (12.2–12.9)	10.5 (10.3–10.8)	9.92 (9.62–10.2)
Unstaged	4.53 (4.37–4.69)	2.79 (2.69–2.90)	4.61 (4.41–4.81)	2.67 (2.55–2.80)
**Grade Differentiation**				
Moderate	1.70 (1.64–1.76)	1.24 (1.20–1.29)	1.13 (1.09–1.18)	1.06 (1.02–1.11)
Poor	3.01 (2.89–3.13)	1.82 (1.75–1.90)	1.91 (1.83–2.00)	1.58 (1.50–1.65)
Undifferentiated	3.09 (2.87–3.33)	2.10 (1.95–2.26)	1.97 (1.78–2.18)	1.65 (1.49–1.83)
Unknown	1.26 (1.21–1.31)	1.08 (1.04–1.13)	2.00 (1.91–2.10)	1.17 (1.11–1.23)
**Surgery (Yes)**	0.228 (0.224–0.232)	0.40 (0.39–0.41)	0.220 (0.216–0.225)	0.40 (0.39–0.41)
**Radiotherapy (Yes)**	1.28 (1.26–1.30)	1.10 (1.08–1.12)	0.81 (0.80–0.83)	1.04 (1.02–1.06)
**Chemotherapy (Yes)**	1.75 (1.72–1.78)	0.80 (0.78–0.82)	1.07 (1.05–1.09)	0.72 (0.70–0.73)

*p* < 0.05 relative to reference unless noted by * *p* ≥ 0.05. Reference categories: Gender (Male), Race (White), Detection Stage (Localized), Grade differentiation (Well), Surgery (No), Radiotherapy (No), and Chemotherapy (No). CI, confidence interval.

**Table 9:** Baseline demographics and clinical characteristics by histology for lung cancers.

Lung	All	Adenocarcinoma	Squamous Cell	Signet Ring
**N**	771,002 (100)	236,024 (30.6)	150,426 (19.5)	1002 (0.1)
**Age (Years) (%)**				
0–14	134 (<0.1)	3 (<0.1)	2 (<0.1)	0 (0)
15–29	1125 (0.1)	191 (0.1)	67 (<0.1)	9 (0.9)
30–49	41,482 (5.4)	15,342 (6.5)	4915 (3.3)	122 (12.2)
50–69	356,243 (46.2)	116,978 (49.6)	68,258 (45.4)	536 (53.5)
70–85	319,649 (41.5)	90,680 (38.4)	69,731 (46.4)	299 (29.8)
>85	52,369 (6.8)	12,830 (5.4)	7453 (5.0)	36 (3.6)
**Mean (SD)**	68.3 (11.4)	67.2 (11.4)	69.2 (10.0)	63.3 (12.8)
**Gender (%)**				
Male	416,219 (54.0)	118,856 (50.4)	97,114 (64.6)	564 (56.3)
Female	354,783 (46.0)	117,168 (49.6)	53,312 (35.4)	438 (43.7)
**Race (%)**				
White	632,947 (82.1)	187,596 (79.5)	123,513 (82.1)	811 (80.9)
Black	87,586 (11.4)	27,778 (11.8)	19,123 (12.7)	118 (11.8)
Other	50,469 (6.5)	20,650 (8.7)	7790 (5.2)	73 (7.3)
**Detection Stage (%)**				
In Situ	781 (0.1)	287 (0.1)	321 (0.2)	0 (0)
Localized	130,847 (17.0)	40,883 (17.3)	33,020 (22.0)	55 (5.5)
Regional	185,572 (24.1)	50,483 (21.4)	53,745 (35.7)	190 (19.0)
Distant	409,261 (53.1)	137,816 (58.4)	57,379 (38.1)	738 (73.7)
Unstaged	44,541 (5.8)	6555 (2.8)	5961 (4.0)	19 (1.9)
**Grade Differentiation (%)**				
Well	31,217 (4.0)	13,206 (5.6)	3664 (2.4)	6 (0.6)
Moderate	102,756 (13.3)	44,436 (18.8)	40,323 (26.8)	71 (7.1)
Poor	187,802 (24.4)	68,573 (29.1)	53,169 (35.3)	396 (39.5)
Undifferentiated	48,057 (6.2)	2123 (0.9)	1539 (1.0)	16 (1.6)
Unknown	401,170 (52.0)	107,686 (45.6)	51,731 (34.4)	513 (51.2)
**Surgery (%)**				
Yes	189,192 (24.5)	66,710 (28.3)	46,764 (31.1)	198 (19.8)
No	581,810 (75.5)	169,314 (71.7)	103,662 (68.9)	804 (80.2)
**Radiotherapy (%)**				
Yes	297,248 (38.6)	92,699 (39.3)	70,553 (46.9)	380 (37.9)
No	473,754 (61.4)	143,325 (60.7)	79,873 (53.1)	622 (62.1)
**Chemotherapy (%)**				
Yes	311,134 (40.4)	101,041 (42.8)	56,174 (37.3)	536 (53.5)
No	459,868 (59.6)	134,983 (57.2)	94,252 (62.7)	466 (46.5)
**Incidence Rate (95% CI)**				
All	60.0 (59.9–60.1)	18.4 (18.3–18.4)	11.7 (11.6–11.7)	7.1 (6.6–7.5) ††
Male	72.7 (72.4–72.9)	20.6 (20.5–20.7)	16.8 (16.7–16.9)	8.6 (7.9–9.4) ††
Female	50.6 (50.4–50.8)	16.8 (16.7–16.9)	7.74 (7.67–7.80)	5.9 (5.4–6.5) ††
**CSS % (95% CI)**				
1-year	46.6 (46.5–46.7)	50.9 (50.7–51.2)	51.2 (50.9–51.6)	33.0 (29.8–36.2)
2-year	31.8 (31.7–32.0)	36.0 (35.8–36.3)	35.2 (34.8–35.5)	20.5 (17.6–23.4)
5-year	20.2 (20.0–20.3)	21.6 (21.4–21.8)	22.5 (22.2–22.9)	9.7 (7.5–12.1)
10-year	14.9 (14.8–15.0)	15.1 (14.9–15.4)	16.2 (15.9–16.5)	4.9 (3.4–6.9)
Median (Months)	10.5	12.5	12.6	5.7
**RS % (95% CI)**				
1-year	44.5 (44.3–44.6)	49.5 (49.2–49.7)	48.8 (48.5–49.2)	31.7 (28.5–34.9)
2-year	29.8 (29.7–29.9)	34.6 (34.4–34.9)	32.6 (32.3–32.9)	19.8 (17.0–22.7)
5-year	18.1 (17.9–18.2)	20.1 (19.9–20.3)	19.5 (19.2–19.8)	9.1 (7.0–11.5)
10-year	12.1 (11.9–12.2)	13.0 (12.8–13.3)	11.6 (11.3–12.0)	4.3 (2.7–6.3)
Median (Months)	9.6	11.7	11.5	5.4

*p* < 0.05 for all comparisons among adenocarcinoma, squamous cell, and signet ring cell comparisons. Incidence rates expressed per 100,000, except **††** (per 10 million). CSS, cause-specific survival; RS, relative survival; CI, confidence interval.

**Table 10:** Derived univariate and multivariable Cox-proportional hazard ratios (HR) of mortality for lung cancers.

Lung	Signet Ring vs. Non-Signet Ring		Signet Ring vs. Adenocarcinoma		Signet Ring vs. Squamous Cell	
HR (95% CI)	Univariate	Multivariable	Univariate	Multivariable	Univariate	Multivariable
**Signet Ring Histology**	1.37 (1.28–1.48)	1.19 (1.10–1.28)	1.48 (1.37–1.59)	1.24 (1.15–1.33)	1.51 (1.40–1.63)	1.17 (1.09–1.26)
**Age (per 10 years)**	1.114 (1.111–1.117)	1.111 (1.108–1.114)	1.070 (1.065–1.074)	1.093 (1.088–1.098)	1.089 (1.082–1.096)	1.072 (1.065–1.079)
**Gender (Female)**	0.827 (0.822–0.832)	0.843 (0.838–0.847)	0.804 (0.796–0.812)	0.83 (0.82–0.84)	0.89 (0.88–0.90)	0.89 (0.87–0.90)
**Race**						
Black	1.09 (1.08–1.10)	1.00 (0.99–1.01) *	1.09 (1.07–1.11)	1.00 (0.98–1.01) *	1.17 (1.15–1.19)	1.01 (0.99–1.03) *
Other	0.94 (0.93–0.95)	0.83 (0.82–0.84)	0.92 (0.91–0.94)	0.77 (0.75–0.78)	1.12 (1.09–1.15)	0.98 (0.95–1.01)*
**Detection Stage**						
In Situ	0.67 (0.57–0.77)	0.59 (0.51–0.68)	0.15 (0.08–0.27)	0.16 (0.09–0.29)	1.01 (0.85–1.20) *	0.73 (0.62–0.87)
Regional	2.28 (2.25–2.30)	2.18 (2.16–2.20)	2.21 (2.17–2.25)	2.26 (2.22–2.31)	2.00 (1.96–2.04)	1.99 (1.95–2.03)
Distant	5.74 (5.68–5.79)	4.48 (4.33–4.53)	6.51 (6.40–6.63)	5.13 (5.03–5.24)	4.91 (4.81–5.00)	3.85 (3.77–3.93)
Unstaged	3.68 (3.62–3.74)	2.32 (2.28–2.35)	3.75 (3.63–3.88)	2.49 (2.41–2.58)	2.89 (2.79–2.99)	1.97 (1.91–2.05)
**Grade Differentiation**						
Moderate	1.62 (1.59–1.65)	1.40 (1.37–1.42)	1.38 (1.34–1.42)	1.29 (1.25–1.33)	0.90 (0.86–0.94)	0.95 (0.91–0.99)
Poor	2.71 (2.66–2.77)	1.79 (1.76–1.83)	2.37 (2.31–2.44)	1.70 (1.65–1.75)	1.06 (1.01–1.10)	1.03 (0.99–1.08) *
Undifferentiated	3.54 (3.46–3.61)	2.02 (1.98–2.07)	2.48 (2.34–2.63)	1.74 (1.65–1.85)	1.21 (1.12–1.30)	1.18 (1.09–1.27)
Unknown	3.61 (3.54–3.67)	1.65 (1.62–1.69)	3.50 (3.41–3.60)	1.54 (1.50–1.59)	1.59 (1.52–1.66)	1.02 (0.98–1.07) *
**Surgery (Yes)**	0.273 (0.271–0.276)	0.453 (0.449–0.457)	0.254 (0.251–0.257)	0.463 (0.456–0.471)	0.296 (0.292–0.301)	0.392 (0.385–0.400)
**Radiotherapy (Yes)**	1.335 (1.328–1.343)	1.034 (1.027–1.040)	1.51 (1.49–1.52)	1.12 (1.10–1.13)	1.43 (1.42–1.45)	0.99 (0.98–1.00) *
**Chemotherapy (Yes)**	1.197 (1.190–1.204)	0.665 (0.660–0.669)	1.25 (1.23–1.26)	0.606 (0.599–0.613)	1.13 (1.11–1.14)	0.65 (0.64–0.66)

*p* < 0.05 relative to reference unless noted by * *p* ≥ 0.05. Reference categories: Gender (Male), Race (White), Detection Stage (Localized), Grade differentiation (Well), Surgery (No), Radiotherapy (No), and Chemotherapy (No). CI, confidence interval.

**Table 11:** Baseline demographics and clinical characteristics by histology for pancreatic cancers.

Pancreatic	All	Adenocarcinoma	Signet Ring
**N**	160,539 (100)	93,923 (58.5)	640 (0.4)
**Age (Years) (%)**			
0–14	61 (<0.1)	0 (0)	0 (0)
15–29	450 (0.3)	66 (0.1)	1 (0.2)
30–49	10,797 (6.7)	6062 (6.5)	52 (8.1)
50–69	68,769 (42.8)	44,493 (47.4)	316 (49.4)
70–85	62,810 (39.1)	36,664 (39.0)	231 (36.1)
>85	17,652 (11.0)	6638 (7.1)	40 (6.3)
**Mean (SD)**	68.9 (12.8)	67.8 (11.7)	66.7 (11.9)
**Gender (%)**			
Male	79,757 (49.7)	48,192 (51.3)	351 (54.8)
Female	80,782 (50.3)	45,731 (48.7)	289 (45.2)
**Race (%)**			
White	128,028 (79.7)	74,880 (79.7)	529 (82.7)
Black	19,779 (12.3)	11,911 (12.7)	63 (9.8)
Other	12,732 (7.9)	7132 (7.6)	48 (7.5)
**Detection Stage (%)**			
In Situ	645 (0.4)	41 (<0.1)	0 (0)
Localized	14,669 (9.1)	6418 (6.8)	17 (2.7)
Regional	43,867 (27.3)	27,826 (29.6)	166 (25.9)
Distant	84,626 (52.7)	55,075 (58.6)	437 (68.3)
Unstaged	16,732 (10.4)	4563 (4.9)	20 (3.1)
**Grade Differentiation (%)**			
Well	8768 (5.5)	3675 (3.9)	1 (0.2)
Moderate	21,250 (13.2)	13,743 (14.6)	32 (5.0)
Poor	22,061 (13.7)	14,872 (15.8)	273 (42.7)
Undifferentiated	1764 (1.1)	582 (0.6)	18 (2.8)
Unknown	106,696 (66.5)	61,051 (65.0)	316 (49.4)
**Surgery (%)**			
Yes	32,963 (20.5)	15,671 (16.7)	134 (20.9)
No	127,576 (79.5)	78,252 (83.3)	506 (79.1)
**Radiotherapy (%)**			
Yes	23,323 (14.5)	16,224 (17.3)	97 (15.2)
No	137,216 (85.5)	77,699 (82.7)	543 (84.8)
**Chemotherapy (%)**			
Yes	66,287 (41.3)	47,158 (50.2)	275 (43.0)
No	94,252 (58.7)	46,765 (49.8)	365 (57.0)
**Incidence Rate (95% CI)**			
All	12.23 (12.18–12.29)	6.97 (6.93–7.02)	4.6 (4.2–4.9) ††
Male	13.89 (13.79–13.98)	8.04 (7.97–8.11)	5.7 (5.1–6.3) ††
Female	10.87 (10.80–10.94)	6.10 (6.04–6.15)	3.7 (3.3–4.1) ††
**CSS % (95% CI)**			
1-year	31.3 (31.0–31.6)	26.6 (26.3–26.9)	20.6 (17.2–24.2)
2-year	17.0 (16.8–17.2)	10.7 (10.5–11.0) *	8.9 (6.5–11.6) *
5-year	9.2 (9.0–9.4)	3.8 (3.6–3.9) *	4.5 (2.8–6.8) *
10-year	6.9 (6.7–7.1)	2.5 (2.4–2.7)	3.0 (1.5–5.3) ^
Median (Months)	5.9	5.6	3.5
**RS % (95% CI)**			
1-year	30.4 (30.2–30.7)	25.9 (25.6–26.2)	20.5 (17.1–24.0)
2-year	16.4 (16.1–16.6)	10.3 (10.1–10.6) *	8.9 (6.6–11.7) *
5-year	8.7 (8.5–8.9)	3.6 (3.4–3.7) *	4.3 (2.6–6.6) *
10-year	6.1 (5.9–6.3)	2.2 (2.1–2.4)	3.1 (1.6–5.5) ^
Median (Months)	5.7	5.4	3.4

*p* < 0.05 for all comparisons between adenocarcinoma and signet ring cell comparisons, unless noted by * *p* ≥ 0.05. Incidence rates expressed per 100,000, except **††** (per 10 million); **^** (not age standardized). CSS, cause-specific survival; RS, relative survival; CI, confidence interval.

**Table 12:** Derived univariate and multivariable Cox-proportional hazard ratios (HR) of mortality for pancreatic cancers.

Pancreatic	Signet Ring vs. Non-Signet Ring		Signet Ring vs. Adenocarcinoma	
HR (95% CI)	Univariate	Multivariable	Univariate	Multivariable
**Signet Ring Histology**	1.27 (1.16–1.39)	1.13 (1.03–1.24)	1.08 (0.98–1.18) *	1.04 (0.95–1.14) *
**Age (per 10 years)**	1.215 (1.209–1.221)	1.170 (1.164–1.176)	1.121 (1.114–1.129) *	1.090 (1.083–1.098)
**Gender (Female)**	1.01 (0.99–1.02) *	0.97 (0.96–0.98)	0.98 (0.97–1.00)	0.96 (0.95–0.97)
**Race**				
Black	1.03 (1.01–1.05)	1.05 (1.03–1.07)	1.07 (1.05–1.10)	1.07 (1.04–1.09)
Other	0.93 (0.91–0.95)	0.95 (0.93–0.97)	1.00 (0.97–1.03) *	0.96 (0.93–0.99)
**Detection Stage**				
In Situ	0.11 (0.09–0.14)	0.13 (0.10–0.17)	0.13 (0.07–0.26)	0.14 (0.07–0.27)
Regional	1.55 (1.51–1.59)	1.70 (1.66–1.74)	1.07 (1.03–1.10)	1.31 (1.27–1.36)
Distant	3.01 (2.94–3.09)	2.64 (2.57–2.70)	2.16 (2.10–2.23)	2.23 (2.16–2.30)
Unstaged	2.46 (2.39–2.54)	1.53 (1.49–1.58)	1.57 (1.51–1.64)	1.24 (1.19–1.30)
**Grade Differentiation**				
Moderate	1.85 (1.79–1.92)	1.86 (1.80–1.92)	1.12 (1.07–1.16)	1.25 (1.20–1.31)
Poor	2.75 (2.66–2.85)	2.42 (2.34–2.51)	1.56 (1.49–1.62)	1.59 (1.53–1.66)
Undifferentiated	2.74 (2.57–2.92)	2.26 (2.12–2.41)	1.63 (1.47–1.80)	1.52 (1.37–1.68)
Unknown	3.11 (3.01–3.20)	1.85 (1.80–1.91)	1.79 (1.73–1.86)	1.34 (1.29–1.39)
**Surgery (Yes)**	0.343 (0.337–0.348)	0.45 (0.44–0.46)	0.42 (0.41–0.43)	0.53 (0.51–0.54)
**Radiotherapy (Yes)**	0.69 (0.68–0.70)	0.99 (0.98–1.01) *	0.58 (0.57–0.59)	0.93 (0.91–0.95)
**Chemotherapy (Yes)**	0.77 (0.76–0.78)	0.67 (0.66–0.68)	0.57 (0.56–0.58)	0.54 (0.53–0.55)

*p* < 0.05 relative to reference unless noted by * *p* ≥ 0.05. Reference categories: Gender (Male), Race (White), Detection Stage (Localized), Grade differentiation (Well), Surgery (No), Radiotherapy (No), and Chemotherapy (No). CI, confidence interval.

**Table 13:** Baseline demographics and clinical characteristics by histology for appendiceal cancers.

Appendiceal	All	Carcinoid	Adenocarcinoma	Signet Ring
**N**	11,456 (100)	4562 (39.8)	1761 (15.4)	669 (5.8)
**Age (Years) (%)**				
0–14	152 (1.3)	151 (3.3)	0 (0)	0 (0)
15–29	990 (8.6)	871 (19.1)	35 (2.0)	4 (0.6)
30–49	3052 (26.6)	1457 (31.9)	333 (18.9)	195 (29.1)
50–69	5114 (44.6)	1680 (36.8)	851 (48.3)	345 (51.6)
70–85	1867 (16.3)	354 (7.8)	455 (25.8)	114 (17.0)
>85	281 (2.5)	49 (1.1)	87 (4.9)	11 (1.6)
**Mean (SD)**	53.9 (17.2)	45.8 (18.0)	61.4 (14.7)	57.7 (12.7)
**Gender (%)**				
Male	5230 (45.7)	1999 (43.8)	931 (52.9)	251 (37.5)
Female	6226 (54.3)	2563 (56.2)	830 (47.1)	418 (62.5)
**Race (%)**				
White	9608 (83.9)	3983 (87.3)	1394 (79.2)	562 (84.0)
Black	1093 (9.5)	370 (8.1)	241 (13.7)	60 (9.0)
Other	755 (6.6)	209 (4.6)	126 (7.2)	47 (7.0)
**Detection Stage (%)**				
In Situ	131 (1.1)	1 (<0.1)	20 (1.1)	1 (0.1)
Localized	4774 (41.7)	2790 (61.2)	661 (37.5)	88 (13.2)
Regional	2620 (22.9)	1087 (23.8)	513 (29.1)	156 (23.3)
Distant	3420 (29.9)	381 (8.4)	527 (29.9)	416 (62.2)
Unstaged	511 (4.5)	303 (6.6)	40 (2.3)	8 (1.2)
**Grade Differentiation (%)**				
Well	3514 (30.7)	1873 (41.1)	242 (13.7)	12 (1.8)
Moderate	2743 (23.9)	427 (9.4)	904 (51.3)	40 (6.0)
Poor	1408 (12.3)	257 (5.6)	370 (21.0)	357 (53.4)
Undifferentiated	176 (1.5)	43 (0.9)	16 (0.9)	39 (5.8)
Unknown	3615 (31.6)	1962 (43.0)	229 (13.0)	221 (33.0)
**Surgery (%)**				
Yes	10,772 (94.0)	4481 (98.2)	1639 (93.1)	588 (87.9)
No	684 (6.0)	81 (1.8)	122 (6.9)	81 (12.1)
**Radiotherapy (%)**				
Yes	228 (2.0)	13 (0.3)	73 (4.1)	20 (3.0)
No	11,228 (98.0)	4549 (99.7)	1688 (95.9)	649 (97.0)
**Chemotherapy (%)**				
Yes	3616 (31.6)	453 (9.9)	723 (41.1)	446 (66.7)
No	7840 (68.4)	4109 (90.1)	1038 (58.9)	223 (33.3)
**Incidence Rate (95% CI)**				
All	8.5 (8.3–8.6) †	3.5 (3.4–3.6) †	1.32 (1.26–1.38) †	5.0 (4.6–5.4) ††
Male	8.2 (8.0–8.4) †	3.1 (2.9–3.2) †	1.53 (1.43–1.63) †	4.2 (3.8–4.8) ††
Female	8.8 (8.6–9.1) †	3.9 (3.7–4.0) †	1.17 (1.09–1.24) †	5.7 (5.2–6.3) ††
**CSS % (95% CI)**				
1-year	87.7 (86.7–88.5)	92.1 (90.2–93.6)	82.7 (80.4–84.7)	74.8 (70.0–78.9)
2-year	78.7 (77.6–79.8)	88.1 (85.9–90.0)	70.7 (68.1–73.2)	55.7 (50.3–60.7)
5-year	63.1 (61.7–64.5)	78.3 (75.3–81.0)	54.0 (50.9–57.0)	34.2 (28.7–39.7)
10-year	53.1 (51.3–54.9)	68.6 (63.2–73.4)	47.2 (43.8–50.5)	24.1 (18.1–30.6)
Median (Months)	-	-	82.1	30.0
**RS % (95% CI)**				
1-year	87.3 (86.2–88.2)	91.1 (88.9–92.9)	81.6 (79.2–83.8)	74.0 (69.1–78.3)
2-year	78.9 (77.6–80.1)	88.0 (85.3–90.2)	70.6 (67.7–73.3)	54.5 (48.9–59.7)
5-year	64.3 (62.5–66.0)	78.8 (74.5–82.4)	55.1 (51.4–58.6)	34.2 (28.4–40.1)
10-year	54.3 (51.7–56.8)	61.8 (54.2–68.5)	48.8 (44.5–52.9)	20.9 (14.4–28.3)
Median (Months)	-	-	107	29.3

*p* < 0.05 for all comparisons among carcinoid, adenocarcinoma, and signet ring cell comparisons. Incidence rates expressed **†** (per 1 million) and **††** (per 10 million). CSS, cause-specific survival; RS, relative survival; CI, confidence interval.

**Table 14:** Derived univariate and multivariable Cox-proportional hazard ratios (HR) of mortality for appendiceal cancers.

Appendiceal	Signet Ring vs. Non-Signet Ring		Signet Ring vs. Carcinoid		Signet Ring vs. Adenocarcinoma	
HR (95% CI)	Univariate	Multivariable	Univariate	Multivariable	Univariate	Multivariable
**Signet Ring Histology**	3.50 (3.16–3.89)	1.50 (1.33–1.68)	8.29 (7.25–9.48)	1.57 (1.33–1.86)	2.00 (1.77–2.26)	0.95 (0.82–1.09) *
**Age (per 10 years)**	1.26 (1.23–1.29)	1.25 (1.22–1.29)	1.44 (1.38–1.50)	1.33 (1.26–1.40)	1.01 (0.97–1.05) *	1.10 (1.05–1.15)
**Gender (Female)**	1.02 (0.95–1.10) *	0.91 (0.85–0.98)	1.30 (1.14–1.49)	1.11 (0.97–1.28)	1.29 (1.15–1.45)	1.06 (0.94–1.20) *
**Race**						
Black	1.29 (1.15–1.44)	1.40 (1.25–1.56)	1.36 (1.10–1.69)	1.57 (1.27–1.95)	1.03 (0.86–1.23) *	1.20 (1.00–1.44) *
Other	1.13 (0.98–1.30) *	0.94 (0.81–1.08) *	1.33 (1.00–1.79) *	0.98 (0.73–1.32) *	0.94 (0.74–1.20) *	0.83 (0.65–1.06) *
**Detection Stage**						
In Situ	0.40 (0.17–0.97)	0.32 (0.13–0.77)	-	-	0.57 (0.14–2.30) *	0.40 (0.10–1.62) *
Regional	2.92 (2.58–3.30)	2.31 (2.04–2.62)	4.18 (3.31–5.29)	3.31 (2.60–4.20)	2.97 (2.40–3.66)	2.83 (2.28–3.51)
Distant	9.24 (8.31–10.3)	6.30 (5.61–7.09)	35.3 (28.6–43.5)	14.7 (11.5–18.7)	11.1 (9.14–13.4)	9.24 (7.45–11.4)
Unstaged	2.94 (2.31–3.75)	2.37 (1.86–3.04)	3.07 (1.76–5.36)	2.55 (1.45–4.48)	3.46 (2.11–5.6)	2.64 (1.60–4.35)
**Grade Differentiation**						
Moderate	2.05 (1.82–2.31)	1.76 (1.56–1.98)	5.41 (3.26–8.98)	2.18 (1.31–3.64)	1.49 (1.13–1.96)	1.43 (1.09–1.88)
Poor	5.64 (5.01–6.36)	3.44 (3.02–3.91)	39.9 (26.2–60.9)	4.92 (3.17–7.65)	4.06 (3.11–5.30)	2.34 (1.77–3.09)
Undifferentiated	5.08 (4.00–6.45)	2.84 (2.23–3.62)	37.2 (22.2–62.3)	5.34 (3.14–9.08)	4.51 (2.97–6.86)	2.34 (1.52–3.59)
Unknown	1.83 (1.63–2.05)	1.83 (1.63–2.05)	7.84 (5.15–12.0)	4.08 (2.67–6.23)	3.55 (2.69–4.68)	2.21 (1.65–2.97)
**Surgery (Yes)**	0.25 (0.22–0.27)	0.49 (0.44–0.55)	0.16 (0.13–0.20)	0.56 (0.45–0.71)	0.25 (0.21–0.30)	0.55 (0.45–0.67)
**Radiotherapy (Yes)**	1.86 (1.56–2.22)	1.19 (0.99–1.43) *	6.11 (4.19–8.90)	1.58 (1.07–2.33)	1.25 (0.97–1.63) *	1.04 (0.80–1.36) *
**Chemotherapy (Yes)**	3.26 (3.03–3.50)	1.37 (1.26–1.48)	9.05 (7.89–10.4)	1.62 (1.36–1.93)	2.13 (1.89–2.41)	0.92 (0.80–1.06) *

*p* < 0.05 relative to reference unless noted by * *p* ≥ 0.05. Reference categories: Gender (Male), Race (White), Detection Stage (Localized), Grade differentiation (Well), Surgery (No), Radiotherapy (No), and Chemotherapy (No). CI, confidence interval.

**Table 15:** Baseline demographics and clinical characteristics by histology for gallbladder/biliary cancers.

Gallbladder/Biliary	All	Adenocarcinoma	Cholangiocarcinoma	Signet Ring
**N**	41,289 (100)	23,923 (57.9)	7483 (18.1)	525 (1.3)
**Age (Years) (%)**				
0–14	13 (<0.1)	1 (<0.1)	2 (<0.1)	0 (0)
15–29	120 (0.3)	59 (0.2)	17 (0.2)	7 (1.3)
30–49	2927 (7.1)	1674 (7.0)	477 (6.4)	50 (9.5)
50–69	16,142 (39.1)	9637 (40.3)	2962 (39.6)	241 (45.9)
70–85	16,720 (40.5)	9867 (41.2)	2989 (39.9)	180 (34.3)
>85	5367 (13.0)	2685 (11.2)	1036 (13.8)	47 (9.0)
**Mean (SD)**	69.7 (13.2)	69.4 (12.8)	69.9 (13.1)	66.5 (13.7)
**Gender (%)**				
Male	17,677 (42.8)	9858 (41.2)	3766 (50.3)	211 (40.2)
Female	23,612 (57.2)	14,065 (58.8)	3717 (49.7)	314 (59.8)
**Race (%)**				
White	32,074 (77.7)	18,724 (78.3)	5757 (76.9)	397 (75.6)
Black	3980 (9.6)	2335 (9.8)	631 (8.4)	65 (12.4)
Other	5235 (12.7)	2864 (12.0)	1095 (14.6)	63 (12.0)
**Detection Stage (%)**				
In Situ	926 (2.2)	389 (1.6)	2 (<0.1)	0 (0)
Localized	8653 (21.0)	5486 (22.9)	986 (13.2)	110 (21.0)
Regional	13,856 (33.6)	8713 (36.4)	2281 (30.5)	224 (42.7)
Distant	13,012 (31.5)	7539 (31.5)	2756 (36.8)	177 (33.7)
Unstaged	4842 (11.7)	1796 (7.5)	1458 (19.5)	14 (2.7)
**Grade Differentiation (%)**				
Well	3503 (8.5)	2341 (9.8)	268 (3.6)	4 (0.8)
Moderate	10,297 (24.9)	7690 (32.1)	941 (12.6)	25 (4.8)
Poor	9102 (22.0)	6190 (25.9)	915 (12.2)	376 (71.6)
Undifferentiated	612 (1.5)	210 (0.9)	43 (0.6)	12 (2.3)
Unknown	17,775 (43.1)	7492 (31.3)	5316 (71.0)	108 (20.6)
**Surgery (%)**				
Yes	21,334 (51.7)	14,545 (60.8)	1516 (20.3)	368 (70.1)
No	19,955 (48.3)	9378 (39.2)	5967 (79.7)	157 (29.9)
**Radiotherapy (%)**				
Yes	6274 (15.2)	3961 (16.6)	1175 (15.7)	104 (19.8)
No	35,015 (84.8)	19,962 (83.4)	6308 (84.3)	421 (80.2)
**Chemotherapy (%)**				
Yes	12,898 (31.2)	7965 (33.3)	2711 (36.2)	203 (38.7)
No	28,391 (68.8)	15,958 (66.7)	4772 (63.8)	322 (61.3)
**Incidence Rate (95% CI)**				
All	2.99 (2.96–3.02)	1.70 (1.67–1.72)	6.2 (6.0–6.3) †	3.9 (3.6–4.2) ††
Male	3.09 (3.04–3.13)	1.69 (1.65–1.72)	7.3 (7.1–7.5) †	3.4 (2.9–3.8) ††
Female	2.94 (2.90–2.97)	1.72 (1.69–1.75)	5.3 (5.1–5.5) †	4.3 (3.9–4.8) ††
**CSS % (95% CI)**				
1-year	47.9 (47.4–48.5)	51.0 (50.3–51.8)	37.0 (35.8–38.2)	45.6 (40.7–50.4)
2-year	32.3 (31.7–32.8)	34.7 (34.0–35.5)	18.6 (17.5–19.6)	31.2 (26.5–36.0)
5-year	20.7 (20.2–21.3)	22.1 (21.4–22.8)	8.6 (7.8–9.5)	16.2 (12.3–20.5)
10-year	17.3 (16.7–17.8)	18.4 (17.7–19.2)	6.5 (5.7–7.4)	12.6 (9.0–16.9)
Median (Months)	11.0	12.5	7.2	10.2
**RS % (95% CI)**				
1-year	45.9 (45.3–46.4)	49.1 (48.3–49.8)	35.1 (33.9–36.3)	42.0 (37.3–46.6)
2-year	30.2 (29.7–30.8)	32.6 (31.9–33.4)	17.1 (16.1–18.1)	27.6 (23.2–32.1)
5-year	18.5 (18.0–19.0)	19.8 (19.2–20.6)	7.4 (6.7–8.2)	13.6 (10.1–17.5)
10-year	14.5 (13.9–15.1)	15.5 (14.7–16.4)	4.9 (4.1–5.7)	10.1 (6.5–14.5)
Median (Months)	10.1	11.6	6.6	9.2

*p* < 0.05 for all comparisons among adenocarcinoma, cholangiocarcinoma, and signet ring cell comparisons. Incidence rates expressed per 100,000, except **†** (per 1 million) and **††** (per 10 million). CSS, cause-specific survival; RS, relative survival; CI, confidence interval.

**Table 16:** Derived univariate and multivariable Cox-proportional hazard ratios (HR) of mortality for gallbladder/biliary cancers.

Gallbladder/Biliary	Signet Ring vs. Non-Signet Ring		Signet Ring vs. Adenocarcinoma		Signet Ring vs. Cholangiocarcinoma	
HR (95% CI)	Univariate	Multivariable	Univariate	Multivariable	Univariate	Multivariable
**Signet Ring Histology**	1.16 (1.05–1.30)	1.12 (1.01–1.25)	1.21 (1.09–1.35)	1.10 (0.99–1.23) *	0.78 (0.70–0.87)	1.06 (0.94–1.20) *
**Age (per 10 years)**	1.17 (1.16–1.18)	1.15 (1.14–1.16)	1.14 (1.13–1.16)	1.15 (1.13–1.16)	1.15 (1.12–1.17)	1.11 (1.09–1.14)
**Gender (Female)**	1.07 (1.04–1.09)	1.05 (1.03–1.08)	1.08 (1.05–1.12)	1.07 (1.03–1.11)	1.19 (1.13–1.25)	1.05 (0.99–1.11) *
**Race**						
Black	1.04 (1.00–1.09) *	1.05 (1.01–1.10)	1.11 (1.05–1.17)	1.09 (1.03–1.15)	1.04 (0.94–1.15) *	1.00 (0.90–1.10) *
Other	0.92 (0.88–0.95)	0.94 (0.91–0.98)	0.92 (0.87–0.96)	0.94 (0.90–0.99)	0.93 (0.86–1.00) *	0.92 (0.85–0.99)
**Detection Stage**						
In Situ	0.17 (0.14–0.20)	0.17 (0.14–0.21)	0.18 (0.13–0.24)	0.17 (0.13–0.23)	0.34 (0.05–2.45) *	0.64 (0.09–4.56)
Regional	1.59 (1.53–1.65)	1.73 (1.66–1.80)	1.56 (1.49–1.64)	1.72 (1.63–1.80)	0.91 (0.84–1.00) *	1.32 (1.21–1.45)
Distant	3.99 (3.84–4.14)	3.28 (3.15–3.43)	4.07 (3.88–4.27)	3.48 (3.30–3.67)	2.00 (1.84–2.18)	2.25 (2.05–2.47)
Unstaged	2.72 (2.59–2.86)	1.45 (1.37–1.52)	2.76 (2.58–2.97)	1.47 (1.36–1.59)	1.40 (1.26–1.55)	1.10 (0.99–1.21) *
**Grade Differentiation**						
Moderate	1.41 (1.34–1.49)	1.30 (1.23–1.38)	1.33 (1.25–1.42)	1.26 (1.18–1.35)	1.08 (0.91–1.27) *	1.08 (0.92–1.28) *
Poor	2.32 (2.19–2.45)	1.92 (1.81–2.03)	2.15 (2.02–2.30)	1.87 (1.75–2.00)	1.49 (1.27–1.76)	1.40 (1.19–1.66)
Undifferentiated	2.25 (2.01–2.52)	1.86 (1.66–2.08)	2.09 (1.76–2.49)	1.86 (1.56–2.22)	1.61 (1.10–2.34)	1.02 (0.69–1.48) *
Unknown	2.76 (2.62–2.91)	1.46 (1.38–1.55)	2.77 (2.59–2.95)	1.51 (1.41–1.62)	2.10 (1.81–2.45)	1.15 (0.98–1.35) *
**Surgery (Yes)**	0.32 (0.31–0.33)	0.43 (0.42–0.45)	0.33 (0.32–0.34)	0.45 (0.43–0.47)	0.39 (0.36–0.41)	0.44 (0.40–0.48)
**Radiotherapy (Yes)**	0.82 (0.79–0.85)	1.02 (0.98–1.06) *	0.82 (0.78–0.85)	1.03 (0.99–1.08) *	0.63 (0.59–0.68)	0.95 (0.88–1.03) *
**Chemotherapy (Yes)**	1.05 (1.03–1.08)	0.74 (0.72–0.76)	1.05 (1.01–1.08)	0.71 (0.69–0.74)	0.73 (0.69–0.77)	0.66 (0.62–0.71)

*p* < 0.05 relative to reference unless noted by * *p* ≥ 0.05. Reference categories: Gender (Male), Race (White), Detection Stage (Localized), Grade differentiation (Well), Surgery (No), Radiotherapy (No), and Chemotherapy (No). CI, confidence interval.

**Table 17:** Baseline demographics and clinical characteristics by histology for breast cancers.

Breast	All	Ductal	Lobular	Signet Ring
**N**	1,185,521 (100)	813,140 (68.6)	245,539 (20.7)	384 (0.03)
**Age (Years) (%)**				
0–14	17 (<0.1)	6 (<0.1)	0 (0)	0 (0)
15–29	6339 (0.5)	4862 (0.6)	508 (0.2)	1 (0.3)
30–49	283,566 (23.9)	202,015 (25.0)	55,048 (22.4)	34 (8.9)
50–69	581,741 (49.1)	399,863 (49.0)	124,886 (50.9)	181 (47.1)
70–85	265,451 (22.4)	176,716 (21.8)	55,945 (22.8)	131 (34.1)
>85	48,407 (4.1)	29,678 (3.7)	9152 (3.7)	37 (9.6)
**Mean (SD)**	60.2 (13.7)	59.7 (13.7)	60.7 (13.1)	67.2 (13.2)
**Gender (%)**				
Male	7059 (0.6)	5958 (0.7)	541 (0.2)	4 (1.0)
Female	1,178,012 (99.4)	807,182 (99.3)	244,998 (99.8)	380 (99.0)
**Race (%)**				
White	956,313 (80.7)	650,557 (80.0)	205,120 (83.5)	338 (88.0)
Black	124,772 (10.5)	88,018 (10.8)	21,734 (8.9)	27 (7.0)
Other	104,436 (8.8)	74,565 (9.2)	18,685 (7.6)	19 (4.9)
**Detection Stage (%)**				
In Situ	231,711 (18.0)	107,886 (13.3)	75,431 (30.7)	1 (0.3)
Localized	589,407 (49.7)	435,830 (53.6)	100,852 (41.1)	151 (39.3)
Regional	301,018 (25.4)	225,149 (27.7)	58,965 (24.0)	110 (28.6)
Distant	63,128 (5.3)	36,705 (4.5)	8696 (3.5)	114 (29.7)
Unstaged	18,257 (1.5)	7570 (0.9)	1595 (0.6)	8 (2.1)
**Grade Differentiation (%)**				
Well	205,026 (17.3)	133,481 (16.4)	44,918 (18.3)	14 (3.6)
Moderate	436,567 (36.8)	305,331 (37.5)	102,616 (41.8)	127 (33.1)
Poor	357,949 (30.2)	287,496 (35.4)	42,394 (17.3)	123 (32.0)
Undifferentiated	28,401 (2.4)	18,932 (2.3)	5421 (2.2)	15 (3.9)
Unknown	157,578 (13.3)	67,900 (8.4)	50,190 (20.4)	105 (27.3)
**Surgery (%)**				
Yes	1,105,455 (93.2)	768,601 (94.5)	233,092 (94.9)	276 (71.9)
No	80,066 (6.8)	44,539 (5.5)	12,447 (5.1)	108 (28.1)
**Radiotherapy (%)**				
Yes	555,742 (46.9)	399,316 (49.1)	105,369 (42.9)	144 (37.5)
No	629,779 (53.1)	413,824 (50.9)	140,170 (57.1)	240 (62.5)
**Chemotherapy (%)**				
Yes	387,251 (32.7)	297,100 (36.5)	61,058 (24.9)	138 (35.9)
No	798,270 (67.3)	516,040 (63.5)	184,481 (75.1)	246 (64.1)
**Incidence Rate (95% CI)**				
All	68.1 (68.0–68.2)	48.6 (48.5–48.7)	12.69 (12.63–12.75)	2.6 (2.3–2.9) ††
Male	11.6 (11.3–11.9) †	9.4 (9.2–9.7) †	6.8 (6.1–7.4) ††	7.5 (2.4–17.6) †††
Female	126.3 (126.0–126.5)	90.4 (90.2–90.6)	23.5 (23.4–23.6)	4.6 (4.2–5.1) ††
**CSS % (95% CI)**				
1-year	96.35 (96.30–96.40)	97.21 (97.16–97.26)	97.96 (97.88–98.04)	88.3 (83.7–91.6)
2-year	93.57 (93.51–93.64)	94.5 (94.4–94.6)	96.1 (96.0–96.2)	80.2 (74.7–84.6)
5-year	87.0 (86.9–87.1)	88.0 (87.9–88.1)	90.1 (89.9–90.3)	65.1 (58.6–70.9)
10-year	80.5 (80.3–80.6)	81.7 (81.5–81.9)	82.6 (82.3–82.8)	55.1 (48.1–61.6)
Median (Months)	-	-	-	-
**RS % (95% CI)**				
1-year	96.93 (96.87–96.99)	97.93 (97.86–98.00)	98.9 (98.8–99.0)	87.5 (82.5–91.2)
2-year	94.7 (94.6–94.8)	95.8 (95.7–95.9)	97.8 (97.7–98.0)	80.3 (73.9–85.2)
5-year	89.2 (89.0–89.3)	90.3 (90.2–90.5)	93.3 (93.0–93.6)	63.4 (55.6–70.2)
10-year	83.0 (82.8–83.3)	84.5 (84.1–84.8)	86.4 (85.8–87.0)	51.6 (41.5–60.8)
Median (Months)	-	-	-	-

*p* < 0.05 for all comparisons among ductal, lobular, and signet ring cell comparisons. Incidence rates expressed per 100,000, except **†** (per 1 million), **††** (per 10 million), and **†††** (per 1 billion). CSS, cause-specific survival; RS, relative survival; CI, confidence interval.

**Table 18:** Derived univariate and multivariable Cox-proportional hazard ratios (HR) of mortality for breast cancers.

Breast	Signet Ring vs. Non-Signet Ring		Signet Ring vs. Ductal		Signet Ring vs. Lobular	
HR (95% CI)	Univariate	Multivariable	Univariate	Multivariable	Univariate	Multivariable
**Signet Ring Histology**	3.58 (3.04–4.20)	1.13 (0.96–1.32) *	3.63 (3.09–4.27)	1.16 (0.98–1.36) *	4.65 (3.96–5.47)	1.19 (1.01–1.40)
**Age (per 10 years)**	1.149 (1.145–1.154)	1.218 (1.213–1.223)	1.094 (1.089–1.099)	1.196 (1.190–1.202)	1.261 (1.248–1.274)	1.274 (1.260–1.288)
**Gender (Male)**	1.61 (1.52–1.70)	1.08 (1.02–1.14)	1.60 (1.51–1.71)	1.11 (1.04–1.18)	1.26 (0.96–1.65) *	0.87 (0.66–1.15) *
**Race**						
Black	1.76 (1.73–1.78)	1.46 (1.44–1.48)	1.84 (1.81–1.87)	1.49 (1.47–1.52)	1.50 (1.44–1.56)	1.50 (1.44–1.57)
Other	0.80 (0.79–0.82)	0.88 (0.86–0.89)	0.84 (0.82–0.86)	0.87 (0.85–0.89)	0.71 (0.67–0.75)	0.89 (0.84–0.95)
**Detection Stage**						
In Situ	0.25 (0.24–0.26)	0.23 (0.22–0.24)	0.29 (0.28–0.31)	0.26 (0.25–0.27)	0.22 (0.20–0.23)	0.21 (0.19–0.23)
Regional	3.43 (3.39–3.48)	3.09 (3.05–3.13)	3.31 (3.26–3.36)	2.97 (2.92–3.02)	3.75 (3.63–3.87)	3.56 (3.44–3.68)
Distant	22.3 (22.0–22.5)	12.5 (12.3–12.7)	21.0 (20.7–21.4)	12.0 (11.8–12.3)	28.3 (27.2–29.4)	16.5 (15.8–17.3)
Unstaged	8.62 (8.40–8.85)	3.91 (3.81–4.03)	6.37 (6.11–6.64)	3.21 (3.07–3.35)	7.52 (6.84–8.27)	3.67 (3.33–4.06)
**Grade Differentiation**						
Moderate	2.31 (2.26–2.36)	1.83 (1.79–1.87)	2.56 (2.49–2.64)	1.99 (1.94–2.05)	1.63 (1.56–1.70)	1.45 (1.39–1.52)
Poor	4.68 (4.58–4.78)	3.21 (3.13–3.28)	5.13 (4.99–5.28)	3.55 (3.44–3.65)	2.55 (2.43–2.67)	2.30 (2.20–2.41)
Undifferentiated	2.48 (2.38–2.57)	3.39 (3.26–3.52)	2.65 (2.53–2.78)	3.63 (3.46–3.81)	0.94 (0.84–1.05) *	2.20 (1.97–2.45)
Unknown	3.70 (3.62–3.79)	2.46 (2.40–2.52)	2.93 (2.83–3.03)	2.62 (2.53–2.71)	1.58 (1.51–1.65)	1.71 (1.63–1.80)
**Surgery (Yes)**	0.118 (0.116–0.119)	0.392 (0.385–0.398)	0.129 (0.127–0.131)	0.39 (0.38–0.40)	0.136 (0.131–0.140)	0.37 (0.35–0.39)
**Radiotherapy (Yes)**	0.696 (0.689–0.703)	0.805 (0.796–0.813)	0.69 (0.68–0.70)	0.79 (0.78–0.80)	0.88 (0.86–0.91)	0.85 (0.83–0.88)
**Chemotherapy (Yes)**	2.22 (2.20–2.24)	1.09 (1.08–1.10)	2.13 (2.10–2.16)	1.06 (1.04–1.08)	2.63 (2.57–2.70)	1.19 (1.16–1.23)

*p* < 0.05 relative to reference unless noted by * *p* ≥ 0.05. Reference categories: Gender (Female), Race (White), Detection Stage (Localized), Grade differentiation (Well), Surgery (No), Radiotherapy (No), and Chemotherapy (No). CI, confidence interval.

**Table 19:** Baseline demographics and clinical characteristics by histology for urinary bladder cancers.

Urinary Bladder	All	Transition Cell	Adenocarcinoma	Signet Ring
**N**	252,104 (100)	237,005 (94.0)	1330 (0.5)	380 (0.2)
**Age (Years) (%)**				
0–14	92 (<0.1)	22 (<0.1)	0 (0)	0 (0)
15–29	844 (0.3)	767 (0.3)	16 (1.2)	1 (0.3)
30–49	14,211 (5.6)	13,146 (5.5)	167 (12.6)	52 (13.7)
50–69	101,475 (40.3)	96,290 (40.6)	528 (39.7)	187 (49.2)
70–85	107,689 (42.7)	101,623 (42.9)	471 (35.4)	122 (32.1)
>85	27,793 (11.0)	25,157 (10.6)	148 (11.1)	18 (4.7)
**Mean (SD)**	69.8 (12.5)	69.7 (12.3)	66.7 (15.1)	64.1 (12.7)
**Gender (%)**				
Male	189,668 (75.2)	180,006 (76.0)	846 (63.6)	269 (70.8)
Female	62,436 (24.8)	56,999 (24.0)	484 (36.4)	111 (29.2)
**Race (%)**				
White	226,912 (90.0)	214,042 (90.3)	1040 (78.2)	316 (83.2)
Black	13,717 (5.4)	12,259 (5.2)	194 (14.6)	45 (11.8)
Other	11,475 (4.6)	10,704 (4.5)	96 (7.2)	19 (5.0)
**Detection Stage (%)**				
In Situ	6454 (2.6)	6386 (2.7)	1 (0.1)	0 (0)
Localized	180,673 (71.7)	176,458 (74.5)	354 (26.6)	52 (13.7)
Regional	46,523 (18.5)	41,382 (17.5)	608 (45.7)	203 (53.4)
Distant	10,761 (4.3)	8002 (3.4)	294 (22.1)	116 (30.5)
Unstaged	7963 (3.1)	4777 (2.0)	73 (5.5)	9 (2.4)
**Grade Differentiation (%)**				
Well	32,449 (12.9)	31,563 (13.3)	64 (4.8)	1 (0.3)
Moderate	71,047 (28.2)	68,936 (29.1)	405 (30.5)	7 (1.8)
Poor	52,063 (20.7)	48,571 (20.5)	456 (34.3)	228 (60.0)
Undifferentiated	58,654 (23.3)	56,559 (23.9)	108 (8.1)	66 (17.4)
Unknown	37,891 (15.0)	31,376 (13.2)	297 (22.3)	78 (20.5)
**Surgery (%)**				
Yes	234,703 (93.1)	224,146 (94.6)	1137 (85.5)	325 (85.5)
No	17,401 (6.9)	12,859 (5.4)	193 (14.5)	55 (14.5)
**Radiotherapy (%)**				
Yes	11,546 (4.6)	9787 (4.1)	171 (12.9)	78 (20.5)
No	240,558 (95.4)	227,218 (95.9)	1159 (87.1)	302 (79.5)
**Chemotherapy (%)**				
Yes	42,371 (16.8)	39,255 (16.6)	307 (23.1)	163 (42.9)
No	209,733 (83.2)	197,750 (83.4)	1023 (76.9)	217 (57.1)
**Incidence Rate (95% CI)**				
All	20.45 (20.37–20.52)	19.04 (18.97–19.12)	1.07 (1.02–1.13) †	3.0 (2.7–3.3) ††
Male	36.1 (35.9–36.2)	33.9 (33.7–34.0)	1.62 (1.52–1.72) †	4.8 (4.2–5.3) ††
Female	8.82 (8.75–7.88)	7.98 (7.92–8.05)	0.67 (0.62–0.73) †	1.5 (1.3–1.8) ††
**CSS % (95% CI)**				
1-year	91.0 (90.8–91.1)	92.6 (92.5–92.7)	73.5 (70.5–76.1)	59.8 (54.0–65.1)
2-year	86.2 (86.0–86.3)	88.1 (88.0–88.3)	63.6 (60.4–66.6)	42.0 (36.1–47.8)
5-year	80.0 (79.7–80.1)	82.0 (81.9–82.2)	49.4 (45.7–52.9)	28.3 (22.7–34.1)
10-year	74.0 (73.8–74.3)	76.1 (75.8–76.3)	41.6 (37.4–45.7)	22.2 (16.7–28.2) ^
Median (Months)	-	-	57.6	15.6
**RS % (95% CI)**				
1-year	90.1 (90.0–90.2)	92.0 (91.8–92.1)	70.2 (67.2–73.0)	57.1 (51.3–62.6)
2-year	85.2 (85.0–85.4)	87.4 (87.2–87.6)	59.4 (56.1–62.6)	38.0 (32.2–43.9)
5-year	78.6 (78.4–78.9)	81.0 (80.7–81.3)	43.3 (39.4–47.1)	24.3 (19.0–29.9)
10-year	71.4 (71.0–71.8)	73.7 (73.3–74.1)	36.5 (32.0–41.1)	16.3 (11.2–22.4) ^
Median (Months)	-	-	42.9	14.4

*p* < 0.05 for all comparisons among transition cell, adenocarcinoma, and signet ring cell comparisons. Incidence rates expressed per 100,000, except **†** (per 1 million) and **††** (per 10 million). CSS, cause-specific survival; RS, relative survival; **^** (not age standardized); CI, confidence interval.

**Table 20:** Derived univariate and multivariable Cox-proportional hazard ratios (HR) of mortality for urinary bladder cancers.

Urinary Bladder	Signet Ring vs. Non-Signet Ring		Signet Ring vs. Transition Cell		Signet Ring vs. Adenocarcinoma	
HR (95% CI)	Univariate	Multivariable	Univariate	Multivariable	Univariate	Multivariable
**Signet Ring Histology**	4.83 (4.25–5.50)	1.56 (1.37–1.78)	5.43 (4.77–6.17)	1.70 (1.49–1.93)	1.74 (1.50–2.03)	1.31 (1.11–1.54)
**Age (per 10 years)**	1.49 (1.48–1.50)	1.47 (1.46–1.48)	1.52 (1.51–1.53)	1.50 (1.48–1.51)	1.15 (1.10–1.21)	1.21 (1.14–1.27)
**Gender (Female)**	1.27 (1.25–1.30)	1.09 (1.07–1.11)	1.19 (1.17–1.22)	1.05 (1.03–1.07)	1.26 (1.09–1.45)	1.11 (0.96–1.29) *
**Race**						
Black	1.70 (1.65–1.75)	1.42 (1.37–1.46)	1.67 (1.61–1.73)	1.44 (1.39–1.50)	1.09 (0.89–1.32) *	1.32 (1.09–1.62)
Other	1.01 (0.96–1.05) *	0.88 (0.84–0.91)	1.00 (0.95–1.04) *	0.88 (0.84–0.92)	0.72 (0.52–0.99)	0.88 (0.64–1.20) *
**Detection Stage**						
In Situ	0.23 (0.15–0.34)	0.24 (0.16–0.36)	0.26 (0.18–0.39)	0.27 (0.18–0.40)	-	-
Regional	6.56 (6.44–6.69)	4.59 (4.50–4.70)	6.66 (6.53–6.80)	4.36 (4.27–4.47)	2.38 (1.92–2.95)	2.22 (1.77–2.80)
Distant	26.9 (26.1–27.6)	19.5 (18.9–20.1)	28.6 (27.7–29.5)	19.4 (18.8–20.1)	8.64 (6.87–10.9)	6.89 (5.34–8.89)
Unstaged	4.17 (3.99–4.35)	2.82 (2.69–2.95)	2.99 (2.82–3.17)	2.27 (2.13–2.42)	2.17 (1.43–3.30)	1.51 (0.98–2.32) *
**Grade Differentiation**						
Moderate	1.58 (1.51–1.65)	1.42 (1.36–1.49)	1.56 (1.49–1.64)	1.43 (1.36–1.50)	1.61 (0.96–2.70) *	1.23 (0.73–2.08) *
Poor	6.23 (5.97–6.50)	2.68 (2.56–2.80)	6.38 (6.10–6.67)	2.84 (2.71–2.97)	3.74 (2.27–6.16)	2.28 (1.37–3.79)
Undifferentiated	6.70 (6.42–7.00)	2.63 (2.51–2.75)	7.21 (6.89–7.54)	2.86 (2.73–3.00)	2.70 (1.58–4.61)	1.93 (1.12–3.32)
Unknown	3.47 (3.31–3.64)	2.05 (1.95–2.15)	2.76 (2.62–2.90)	1.96 (1.86–2.06)	2.47 (1.47–4.13)	1.50 (0.89–2.52) *
**Surgery (Yes)**	0.49 (0.48–0.51)	0.64 (0.62–0.66)	0.63 (0.61–0.65)	0.68 (0.66–0.71)	0.35 (0.29–0.42)	0.55 (0.44–0.68)
**Radiotherapy (Yes)**	5.32 (5.18–5.45)	1.27 (1.24–1.31)	5.80 (5.64–5.96)	1.28 (1.25–1.32)	1.88 (1.57–2.24)	1.17 (0.97–1.41) *
**Chemotherapy (Yes)**	2.19 (2.15–2.24)	0.97 (0.94–0.99)	2.26 (2.22–2.31)	0.99 (0.96–1.01) *	1.78 (1.54–2.05)	0.93 (0.79–1.10) *

*p* < 0.05 relative to reference unless noted by * *p* ≥ 0.05. Reference categories: Gender (Male), Race (White), Detection Stage (Localized), Grade differentiation (Well), Surgery (No), Radiotherapy (No), and Chemotherapy (No). CI, confidence interval.

**Table 21:** Baseline demographics and clinical characteristics by histology for small bowel cancers.

Small Bowel	All	Carcinoid	Adenocarcinoma	Signet Ring
**N**	25,899 (100)	13,837 (53.4)	6111 (23.6)	327 (1.3)
**Age (Years) (%)**				
0–14	19 (0.1)	4 (<0.1)	0 (0)	0 (0)
15–29	248 (1.0)	113 (0.8)	42 (0.7)	5 (1.5)
30–49	3912 (15.1)	2091 (15.1)	794 (13.0)	55 (16.8)
50–69	12,530 (48.4)	7337 (53.0)	2533 (41.4)	157 (48.0)
70–85	7567 (29.2)	3725 (26.9)	2129 (34.8)	90 (27.5)
>85	1623 (6.3)	567 (4.2)	613 (10.0)	20 (6.1)
**Mean (SD)**	63.6 (14.1)	62.5 (13.1)	66.4 (14.3)	62.7 (14.3)
**Gender (%)**				
Male	13,517 (52.2)	7074 (51.1)	3244 (53.1)	185 (56.6)
Female	12,382 (47.8)	6763 (48.9)	2867 (46.6)	142 (43.4)
**Race (%)**				
White	20,207 (78.0)	11,066 (80.0)	4521 (74.0)	263 (80.4)
Black	4180 (16.1)	2305 (16.7)	1136 (18.6)	39 (11.9)
Other	1512 (5.8)	466 (3.4)	454 (7.4)	25 (7.6)
**Detection Stage (%)**				
In Situ	188 (0.7)	10 (0.1)	32 (0.5)	0 (0)
Localized	8057 (31.1)	4662 (33.7)	1237 (20.2)	33 (10.1)
Regional	8590 (33.2)	4980 (36.0)	2100 (34.4)	142 (43.4)
Distant	7177 (27.7)	3359 (24.3)	2227 (36.4)	132 (40.4)
Unstaged	1887 (7.3)	826 (6.0)	515 (8.4)	20 (6.1)
**Grade Differentiation (%)**				
Well	6349 (24.5)	5293 (38.3)	453 (7.4)	0 (0)
Moderate	4867 (18.8)	1235 (8.9)	2559 (41.9)	16 (4.9)
Poor	3101 (12.0)	185 (1.3)	1880 (30.8)	229 (70.0)
Undifferentiated	515 (2.0)	52 (0.4)	62 (1.0)	9 (2.8)
Unknown	11,067 (42.7)	7072 (51.1)	1157 (18.9)	73 (22.3)
**Surgery (%)**				
Yes	20,068 (77.5)	11,733 (84.8)	3671 (60.1)	219 (67.0)
No	5831 (22.5)	2104 (15.2)	2440 (39.9)	108 (33.0)
**Radiotherapy (%)**				
Yes	993 (3.8)	134 (1.0)	604 (9.9)	34 (10.4)
No	24,906 (96.2)	13,703 (99.0)	5507 (90.1)	293 (89.6)
**Chemotherapy (%)**				
Yes	5280 (20.4)	724 (5.2)	2452 (40.1)	160 (48.9)
No	20,619 (79.6)	13,113 (94.8)	3659 (59.9)	167 (51.1)
**Incidence Rate (95% CI)**				
All	2.11 (2.08–2.13)	1.12 (1.11–1.14)	5.2 (5.1–5.4) †	2.6 (2.4–2.9) ††
Male	2.49 (2.45–2.53)	1.29 (1.26–1.32)	6.4 (6.2–6.6) †	3.3 (2.9–3.8) ††
Female	1.81 (1.78–1.84)	0.99 (0.97–1.01)	4.3 (4.1–4.4) †	2.1 (1.8–2.4) ††
**CSS % (95% CI)**				
1-year	80.9 (80.4–81.5)	94.9 (94.4–95.3)	56.1 (54.7–57.5)	54.2 (47.7–60.2)
2-year	74.5 (73.9–75.1)	92.4 (91.8–92.9)	41.8 (40.3–43.2)	28.6 (22.8–34.8)
5-year	64.8 (64.1–65.5)	85.0 (84.2–85.8)	27.4 (26.0–28.9)	15.5 (10.7–21.0)
10-year	56.1 (55.2–57.0)	74.7 (73.3–75.9)	23.1 (21.7–24.6)	13.8 (9.2–19.3)
Median (Months)	-	-	16.2	14
**RS % (95% CI)**				
1-year	78.7 (78.1–79.3)	93.2 (92.6–93.8)	53.7 (52.3–55.1)	50.8 (44.4–56.8)
2-year	72.5 (71.8–73.2)	91.0 (90.2–91.7)	39.6 (38.2–41.0)	25.9 (20.4–31.7)
5-year	63.4 (62.5–64.2)	84.3 (83.1–85.5)	26.1 (24.6–27.5)	13.8 (9.5–19.0)
10-year	53.2 (51.9–54.5)	71.8 (69.5–73.9)	21.4 (19.8–23.2)	10.2 (6.3–15.2)
Median (Months)	-	-	14.4	13

*p* < 0.05 for all comparisons among carcinoid, adenocarcinoma, and signet ring cell comparisons. Incidence rates expressed per 100,000, except **†** (per 1 million) and **††** (per 10 million). CSS, cause-specific survival; RS, relative survival; CI, confidence interval.

**Table 22:** Derived univariate and multivariable Cox-proportional hazard ratios (HR) of mortality for small bowel cancers.

Small Bowel	Signet Ring vs non-Signet Ring		Signet Ring vs Carcinoid		Signet Ring vs Adenocarcinoma	
HR (95% CI)	Univariate	Multivariable	Univariate	Multivariable	Univariate	Multivariable
**Signet Ring Histology**	4.10 (3.59–4.69)	1.64 (1.43–1.88)	12.9 (11.2–14.9)	4.47 (3.57–5.60)	1.35 (1.18–1.54)	1.23 (1.06–1.41)
**Age (per 10 years)**	1.31 (1.29–1.34)	1.32 (1.30–1.35)	1.36 (1.32–1.41)	1.46 (1.41–1.51)	1.20 (1.17–1.23)	1.15 (1.12–1.18)
**Gender (Female)**	0.97 (0.93-1.02) *	0.94 (0.90–0.98)	1.06 (0.98–1.15)	0.99 (0.92–1.08) *	1.02 (0.95–1.09) *	1.00 (0.94–1.07) *
**Race**						
Black	1.02 (0.96-1.08) *	1.10 (1.03–1.17)	0.75 (0.66–0.85)	1.00 (0.88-1.13) *	1.01 (0.93–1.10) *	1.02 (0.93–1.11) *
Other	1.15 (1.05-1.27)	0.99 (0.90–1.08) *	0.71 (0.54–0.92)	0.73 (0.56–0.96)	0.97 (0.85–1.10) *	0.88 (0.77–1.00) *
**Detection Stage**						
In Situ	1.09 (0.74-1.61) *	0.91 (0.61–1.34) *	1.94 (0.27-13.9) *	1.41 (0.20-10.1) *	0.88 (0.46–1.71) *	0.69 (0.36–1.34) *
Regional	2.20 (2.05–2.36)	2.06 (1.91–2.21)	2.78 (2.39–3.23)	2.99 (2.57–3.49)	1.79 (1.61–2.00)	2.03 (1.81–2.27)
Distant	5.91 (5.53–6.32)	4.62 (4.31–4.96)	9.64 (8.38–11.1)	8.90 (7.71–10.3)	5.10 (4.58–5.67)	4.53 (4.03–5.09)
Unstaged	3.35 (3.02–3.71)	1.93 (1.73–2.15)	2.94 (2.32–3.73)	1.82 (1.42–2.32)	3.72 (3.20–4.34)	1.95 (1.66–2.28)
**Grade Differentiation**						
Moderate	3.96 (3.63–4.32)	3.50 (3.20–3.82)	1.70 (1.42–2.03)	1.46 (1.22–1.75)	1.22 (1.05–1.42)	1.12 (0.96–1.30) *
Poor	8.14 (7.46–8.88)	5.58 (5.08–6.11)	10.5 (8.95–12.3)	2.94 (2.34–3.70)	1.77 (1.52–2.06)	1.50 (1.28–1.75)
Undifferentiated	5.95 (5.17–6.85)	5.11 (4.43–5.89)	11.4 (8.2–15.8)	4.64 (3.32–6.50)	2.13 (1.55–2.92)	2.23 (1.62–3.07)
Unknown	2.07 (1.90–2.24)	1.63 (1.50–1.77)	1.54 (1.38–1.72)	1.45 (1.29–1.62)	2.54 (2.17–2.98)	1.17 (0.99–1.37) *
**Surgery (Yes)**	0.28 (0.26–0.29)	0.36 (0.34–0.38)	0.42 (0.38–0.46)	0.47 (0.42–0.53)	0.28 (0.26–0.30)	0.40 (0.37–0.43)
**Radiotherapy (Yes)**	2.93 (2.71–3.18)	1.38 (1.27–1.50)	4.42 (3.55–5.51)	1.53 (1.22–1.92)	1.07 (0.97–1.18) *	1.10 (0.99–1.22) *
**Chemotherapy (Yes)**	2.69 (2.57–2.82)	1.31 (1.24–1.38)	4.66 (4.20–5.17)	1.73 (1.53–1.96)	0.94 (0.88–1.01) *	0.66 (0.62–0.71)

*p* < 0.05 relative to reference unless noted by * *p* ≥ 0.05. Reference categories: Gender (Male), Race (White), Detection Stage (Localized), Grade differentiation (Well), Surgery (No), Radiotherapy (No), and Chemotherapy (No). CI, confidence interval.

**Table 23:** Baseline demographics and clinical characteristics by histology for ovarian cancers.

Ovarian	All	Papillary Serous Cystadeno.	Adenocarcinoma	Signet
**N**	104,705 (100)	25,411 (24.3)	11,844 (11.3)	186 (0.2)
**Age (Years) (%)**				
0–14	645 (0.6)	6 (<0.1)	2 (<0.1)	0 (0)
15–29	3906 (3.7)	493 (1.9)	58 (0.5)	3 (1.6)
30–49	20,498 (19.6)	4802 (18.9)	952 (8.1)	34 (18.3)
50–69	45,891 (43.8)	12,533 (49.3)	4351 (36.7)	86 (46.2)
70–85	27,087 (25.9)	6782 (26.7)	4903 (41.4)	51 (27.4)
>85	6678 (6.4)	795 (3.1)	1578 (13.3)	12 (6.5)
**Mean (SD)**	60.7 (16.6)	61.0 (14.0)	69.7 (13.6)	62.7 (14.7)
**Race (%)**				
White	87,315 (83.4)	22,197 (87.4)	9846 (83.1)	153 (82.3)
Black	8499 (8.1)	1674 (6.6)	1265 (10.7)	16 (8.6)
Other	8891 (8.5)	1540 (6.1)	733 (6.2)	17 (9.1)
**Detection Stage (%)**				
In Situ	621 (0.6)	14 (0.1)	28 (0.2)	0 (0)
Localized	23,494 (22.4)	2946 (11.6)	444 (3.7)	9 (4.8)
Regional	9969 (9.5)	1953 (7.7)	611 (5.2)	8 (4.3)
Distant	64,289 (61.4)	20,088 (79.1)	9866 (83.3)	161 (86.6)
Unstaged	6332 (5.0)	410 (1.6)	895 (7.6)	8 (4.3)
**Grade Differentiation (%)**				
Well	7167 (6.8)	1152 (4.6)	162 (1.4)	0 (0)
Moderate	12,908 (12.3)	3807 (15.0)	569 (4.8)	9 (4.8)
Poor	30,025 (28.7)	10,154 (40.0)	3002 (25.3)	68 (36.6)
Undifferentiated	12,532 (12.0)	3583 (14.1)	322 (2.7)	4 (2.2)
Unknown	42,073 (40.2)	6715 (26.4)	7789 (65.8)	105 (56.5)
**Surgery (%)**				
Yes	82,018 (78.3)	23,452 (92.3)	4154 (35.1)	97 (52.2)
No	22,687 (21.7)	1959 (7.7)	7690 (64.9)	89 (47.8)
**Radiotherapy (%)**				
Yes	1567 (1.5)	266 (1.0)	200 (1.7)	1 (0.5)
No	103,138 (98.5)	25,145 (99.0)	11,644 (98.3)	185 (99.5)
**Chemotherapy (%)**				
Yes	63,158 (60.3)	18,316 (72.1)	7101 (60.0)	114 (61.3)
No	41,547 (39.7)	7095 (27.9)	4743 (40.0)	72 (38.7)
**Incidence Rate (95% CI) ^**	12.5 (12.4–12.6)	2.75 (2.71–2.79)	1.40 (1.38–1.43)	2.0 (1.7–2.4) ††
**CSS % (95% CI)**				
1-year	72.8 (72.5–73.2)	83.0 (82.4–83.5)	50.7 (49.6–51.7)	41.7 (33.1–50.0)
2-year	70.0 (60.6–61.3)	68.0 (67.3–68.7)	36.9 (35.9–38.0)	25.4 (18.1–33.5)
5-year	40.6 (40.1–40.9)	38.2 (37.5–39.0)	19.2 (18.3–20.2)	10.8 (6.0–17.1)
10-year	30.4 (30.0–30.8)	23.3 (22.6–24.1)	12.9 (12.1–13.8)	6.6 (2.9–12.4)
Median (Months)	39.2	41.9	12.5	7.9
**RS % (95% CI)**				
1-year	72.0 (71.7–72.3)	83.1 (82.4–83.7)	49.1 (48.1–50.2)	40.1 (31.7–48.4)
2-year	60.6 (60.2–60.9)	68.6 (67.8–69.4)	35.8 (34.7–36.8)	24.2 (16.9–32.1)
5-year	40.6 (40.2–41.0)	39.3 (38.5–40.1)	18.5 (17.6–19.4)	9.9 (5.3–16.1)
10-year	30.7 (30.2–31.2)	24.5 (23.6–25.4)	12.3 (11.4–13.2)	7.1 (3.1–13.4)
Median (Months)	38.9	43.3	11.3	7.4

*p* < 0.05 for all comparisons among papillary serous cystoadenocarcinoma, adenocarcinoma, and signet ring cell comparisons. Incidence rates (**^** indicates calculated among female population) expressed per 100,000 except **††** (per 10 million). CSS, cause-specific survival; RS, relative survival; CI, confidence interval.

**Table 24:** Derived univariate and multivariable Cox-proportional hazard ratios (HR) of mortality for ovarian cancers.

Ovarian	Signet Ring vs. non-Signet Ring		Signet Ring vs. Papillary Serous Cystadenocarcinoma		Signet Ring vs. Adenocarcinoma	
HR (95% CI)	Univariate	Multivariable	Univariate	Multivariable	Univariate	Multivariable
**Signet Ring Histology**	2.95 (2.47–3.51)	1.56 (1.31–1.86)	2.99 (2.51–3.56)	1.87 (1.56–2.23)	1.15 (0.96-1.37) *	1.30 (1.09–1.56)
**Age (per 10 years)**	1.52 (1.51–1.53)	1.29 (1.28–1.30)	1.38 (1.36–1.39)	1.27 (1.26–1.29)	1.34 (1.31–1.36)	1.16 (1.13–1.18)
**Race**						
Black	1.17 (1.13–1.20)	1.20 (1.16–1.24)	1.08 (1.01–1.15)	1.18 (1.11–1.26)	1.24 (1.16–1.34)	1.21 (1.13-1.31)
Other	0.72 (0.70–0.75)	0.94 (0.91–0.98)	0.83 (0.77–0.89)	0.94 (0.88–1.01) *	0.86 (0.78–0.94)	0.95 (0.86-1.04) *
**Detection Stage**						
In Situ	0.34 (0.22–0.54)	0.35 (0.22–0.55)	-	-	-	-
Regional	4.70 (4.45–4.97)	3.70 (3.49–3.91)	4.32 (3.79–4.91)	3.31 (2.91–3.77)	4.43 (3.49–5.62)	4.00 (3.14–5.10)
Distant	13.2 (12.6–13.7)	9.01 (8.61–9.43)	12.0 (10.8–13.3)	8.90 (7.99–9.92)	9.62 (7.79–11.9)	7.56 (6.07–9.40)
Unstaged	13.0 (12.3–13.7)	5.17 (4.88–5.48)	8.78 (7.45–10.3)	5.56 (4.70–6.57)	9.80 (7.59–11.9)	5.34 (4.23–6.74)
**Grade Differentiation**						
Moderate	2.86 (2.69–3.05)	1.76 (1.65–1.88)	2.54 (2.28–2.82)	1.99 (1.79–2.22)	3.16 (2.33–4.30)	1.47 (1.08–2.01)
Poor	5.17 (4.87–5.49)	2.08 (1.96–2.21)	3.20 (2.89–3.53)	2.14 (1.93–2.37)	3.30 (2.46–4.42)	1.35 (1.00–1.81) *
Undifferentiated	4.69 (4.41–5.00)	1.98 (1.86–2.11)	2.99 (2.69–3.33)	2.03 (1.83–2.26)	3.22 (2.34–4.43)	1.43 (1.03–1.97)
Unknown	4.43 (4.17–4.70)	1.81 (1.70–1.92)	1.93 (1.74–2.14)	1.75 (1.58–1.94)	5.97 (4.47–7.99)	1.46 (1.08–1.97)
**Surgery (Yes)**	0.21 (0.20-0.22)	0.36 (0.35-0.37)	0.30 (0.28-0.31)	0.38 (0.36-0.41)	0.33 (0.31-0.35)	0.47 (0.44-0.49)
**Radiotherapy (Yes)**	1.34 (1.26-1.43)	1.19 (1.12-1.27)	1.11 (0.96-1.29)	0.99 (0.85-1.15) *	1.05 (0.90-1.23) *	1.05 (0.89-1.22) *
**Chemotherapy (Yes)**	1.64 (1.61-1.67)	0.91 (0.89-0.93)	1.98 (1.90-2.06)	1.08 (1.04-1.13)	0.67 (0.64-0.71)	0.59 (0.56-0.62)

*p* < 0.05 relative to reference unless noted by * *p* ≥ 0.05. Reference categories: Race (White), Detection Stage (Localized), Grade differentiation (Well), Surgery (No), Radiotherapy (No), and Chemotherapy (No). CI, confidence interval.

**Table 25:** Baseline demographics and clinical characteristics by histology for prostate cancers.

Prostate	All	Adenocarcinoma	Signet Ring
**N**	999,669 (100)	959,899 (96.0)	152 (0.02)
**Age (Years) (%)**			
0–14	44 (<0.1)	0 (0) *	0 (0) *
15–29	43 (<0.1)	10 (<0.1) *	0 (0) *
30–49	27,207 (2.7)	26,290 (2.7) *	4 (2.6) *
50–69	578,769 (57.9)	563,449 (58.7) *	83 (54.6) *
70–85	356,646 (35.7)	340,627 (35.5) *	60 (39.5) *
>85	36,960 (3.7)	29,523 (3.1) *	5 (3.3) *
**Mean (SD)**	67.1 (9.5)	66.8 (9.3) *	68.5 (9.8) *
**Race (%)**			
White	796,772 (79.7)	764,998 (79.7)	107 (70.4)
Black	148,696 (14.9)	143,025 (14.9)	31 (0.4)
Other	54,201 (5.4)	51,876 (5.4)	14 (9.2)
**Detection Stage (%)**			
In Situ	155 (<0.1)	75 (<0.1)	0 (0)
Localized/Regional	840,596 (84.1)	822,046 (85.6)	114 (75.0)
Distant	48,394 (4.8)	40,274 (4.2)	20 (13.2)
Unstaged	110,524 (11.1)	97,504 (10.2)	18 (11.8)
**Grade Differentiation (%)**			
Well	67,068 (6.7)	66,219 (6.9)	0 (0)
Moderate	503,255 (50.3)	494,704 (51.5)	11 (7.2)
Poor	365,431 (36.6)	358,221 (37.3)	137 (90.1)
Undifferentiated	2977 (0.3)	2541 (0.3)	1 (0.7)
Unknown	60,938 (6.1)	38,214 (4.0)	3 (2.0)
**Surgery (%)**			
Yes	439,779 (44.0)	426,321 (44.4) *	71 (46.7) *
No	559,890 (56.0)	533,578 (55.6) *	81 (53.3) *
**Radiotherapy (%)**			
Yes	343,522 (34.4)	336,965 (35.1) *	50 (32.9) *
No	656,147 (65.6)	622,934 (64.9) *	102 (67.1) *
**Chemotherapy (%)**			
Yes	8234 (0.8)	7086 (0.7) *	3 (2.0) *
No	991,435 (99.2)	952,813 (99.3) *	149 (98.0) *
**Incidence Rate (95% CI) ^**	141.2 (140.9–141.5)	134.1 (133.8–134.3)	2.0 (1.7–2.4) ††
**CSS % (95% CI)**			
1-year	97.83 (97.79–97.86)	98.49 (98.45–98.52)	96.0 (90.7–98.3)
2-year	96.07 (96.03–96.12)	96.92 (96.87–96.96)	92.4 (84.0–96.5)
5-year	92.33 (92.26–92.39)	93.35 (93.28–93.42)	83.6 (72.5–90.5)
10-year	87.1 (87.0–87.2)	88.1 (88.0–88.2)	69.8 (56.4–79.8)
Median (Months)	-	-	-
**RS (Months) (95% CI)**			
1-year	99.00 (98.95–99.04)	99.70 (99.67–99.74)	94.9 (88.1–97.8)
2-year	98.37 (98.30–98.43)	99.35 (99.29–99.40)	88.5 (76.0–94.7)
5-year	97.4 (97.3–97.5)	98.7 (98.6–98.8)	79.4 (64.1–88.7)
10-year	95.8 (95.1–96.0)	97.2 (96.9–97.5)	65.9 (46.8–79.5)
Median (Months)	-	-	-

*p* < 0.05 for all comparisons between adenocarcinoma and signet ring cell comparisons, unless noted by * *p* ≥ 0.05. Incidence rates (**^** indicates calculated among male population) expressed per 100,000 except **††** (per 10 million). CSS, cause-specific survival; RS, relative survival; CI, confidence interval.

**Table 26:** Derived univariate and multivariable Cox-proportional hazard ratios (HR) of mortality for prostate cancers.

Prostate	Signet Ring vs. non-Signet Ring		Signet Ring vs. Adenocarcinoma	
HR (95% CI)	Univariate	Multivariable	Univariate	Multivariable
**Signet Ring Histology**	2.41 (1.71–3.39)	1.17 (0.83–1.65) *	2.63 (1.87–3.70)	1.16 (0.83–1.63) *
**Age (per 10 years)**	2.11 (2.09–2.12)	1.67 (1.65–1.68)	2.01 (1.99–2.02)	1.67 (1.66–1.69)
**Race**				
Black	1.27 (1.25–1.29)	1.28 (1.26–1.31)	1.30 (1.27–1.32)	1.31 (1.29–1.33)
Other	0.99 (0.97–1.02) *	0.77 (0.75–0.79)	0.98 (0.95–1.01) *	0.76 (0.74–0.79)
**Detection Stage**				
In Situ	1.21 (0.67–2.18) *	0.56 (0.31–1.01) *	1.17 (0.52–2.60) *	0.60 (0.27–1.34) *
Distant	27.4 (27.0–27.8)	13.6 (13.3–13.8)	26.7 (26.2–27.1)	14.1 (13.9–14.4)
Unstaged	3.02 (2.97–3.07)	2.62 (2.57–2.66)	2.66 (2.62–2.71)	2.56 (2.51–2.60)
**Grade Differentiation**				
Moderate	1.03 (0.99–1.08) *	1.37 (1.31–1.42)	1.04 (0.99–1.08) *	1.35 (1.30–1.42)
Poor	3.21 (3.09–3.34)	3.39 (3.26–3.53)	3.26 (3.14–3.39)	3.38 (3.25–3.52)
Undifferentiated	8.82 (8.22–9.47)	5.94 (5.54–6.38)	7.85 (7.27–8.47)	5.28 (4.89–5.70)
Unknown	8.87 (8.52–9.24)	3.87 (3.72–4.04)	6.50 (6.23–6.79)	3.37 (3.22–3.52)
**Surgery (Yes)**	0.486 (0.479–0.492)	0.72 (0.71–0.73)	0.524 (0.517–0.531)	0.73 (0.72–0.74)
**Radiotherapy (Yes)**	0.79 (0.78–0.80)	0.84 (0.83–0.86)	0.84 (0.83–0.85)	0.85 (0.83–0.86)
**Chemotherapy (Yes)**	7.21 (6.96–7.45)	2.30 (2.22–2.38)	6.99 (6.73–7.26)	2.24 (2.15–2.33)

*p* < 0.05 relative to reference unless noted by * *p* ≥ 0.05. Reference categories: Race (White), Detection Stage (Localized/Regional), Grade differentiation (Well), Surgery (No), Radiotherapy (No), and Chemotherapy (No). CI, confidence interval.

## Supplementary Materials

The following are available online at https://www.mdpi.com/2072-6694/12/6/1544/s1, Figure S1: Jointpoint analysis for gastric and colon cancers (1975–2016), Table S1: Exclusion criteria and counts of all cases and signet ring cell cases, Table S2. Count of all cases and signet cell cases by SEER cancer registry for all included cases. Table S3: Variables in analysis, Table S4: Breakdown of SRCC cases in SEER (1975–2016), both analyzed and not analyzed.
