# Supplementary material for: Epidemiology of Signet Ring Cell Adenocarcinomas
Source: Cancers (Basel). 2020 Jun 11;12(6):1544. doi: 10.3390/cancers12061544 (PMC7352645; doi:10.3390/cancers12061544)
Supplement: Supplementary file 1 [file cancers-12-01544-s001.pdf]

cancers

MDPI

Supplementary Material

Epidemiology of Signet Ring Cell Adenocarcinomas

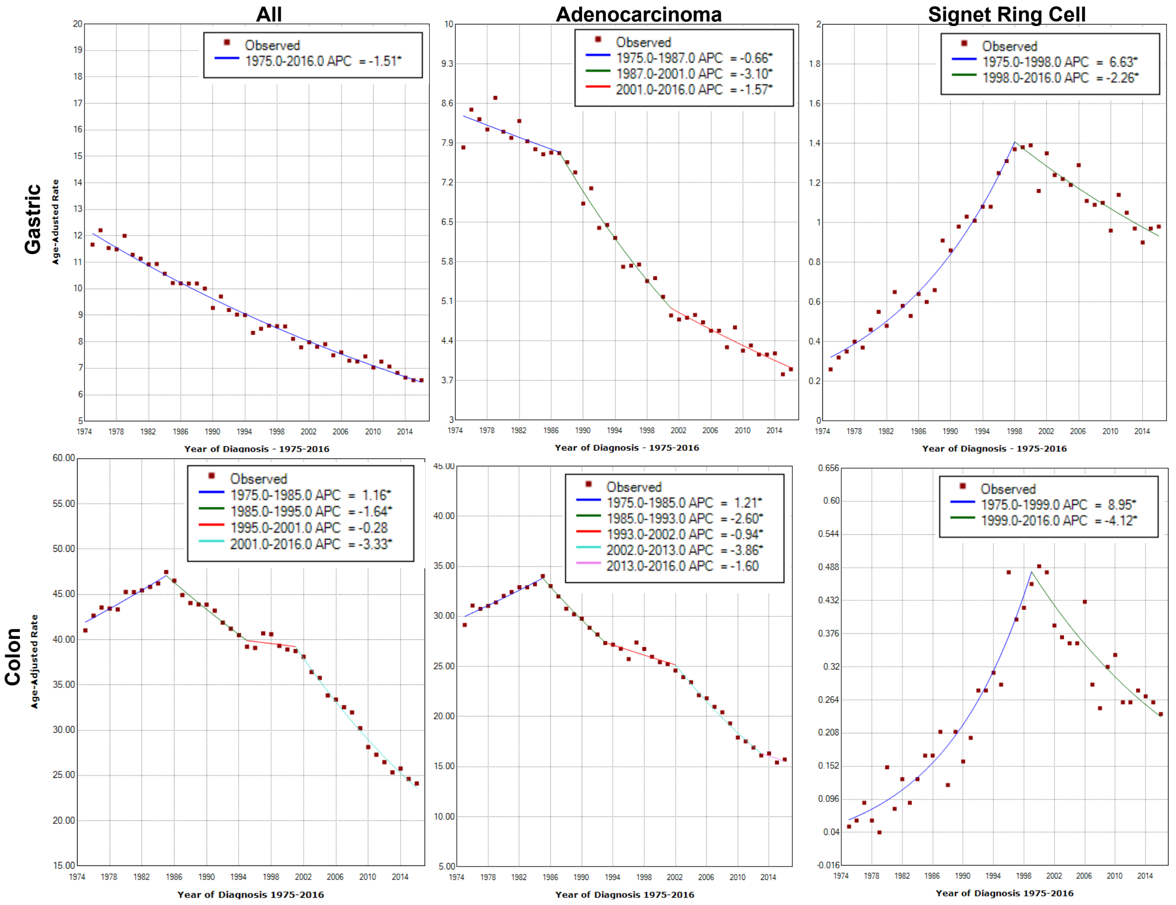

Figure S1. Jointpoint analysis for gastric and colon cancers (1975–2016), using SEER 9 data, with adenocarcinoma and signet ring cell data subsets, using Jointpoint Trend Analysis Software. The WHO formalized the definition of the signet ring cell cancer histotype for gastric cancer in 1990. Analysis of signet ring cell data is subsequently limited to cases after 1992 to limit potential misclassification of the signet ring cell phenotype. * Indicates that the Annual Percent Change (APC) is significantly difference from zero at the p = 0.05 level. Age-adjusted rates are expressed per 100,000 population.

Table S1. Exclusion criteria and counts of all cases and signet ring cell cases.

Data Processing Scheme	Count (All)	Count (Signet Ring)
Initial count of all non-blood borne cancer cases.	9,608,247	41,847
Drop if year diagnosis ≤1991 (year_dx ≤ 1991).	8,229,651	38,768
Drop if cancer is not patient's primary or first (seq_num ≥ 2).	6,587,624	31,892
Drop if cause-specific death classification unknown/missing (vsrtsadx = 8).	6,545,624	31,420
Drop if survival months is unknown (srv_time_mon = 9999).	6,468,886	31,357
Drop if age_dx is unknown (age_dx = 999).	6,467,693	31,356
Drop if race unknown (race1v = 99).	6,387,182	31,298
Drop if surgery is unknown (surgprif, ss_surg = 99).	6,350,139	31,187
Drop if any variable in Table 3 missing data.	6,350,139	31,187

Table S2. Count of all cases and signet cell cases by SEER cancer registry for all included cases.

SEER Registry	SEER #	Years of Diagnosis	Count (All)	Count (Signet Ring)
San Francisco-Oakland	SEER 9	1975-2016	389,446	2,037
Connecticut	SEER 9	1975-2016	385,805	1,954
Metropolitan Detroit	SEER 9	1975-2016	425,465	1,870
Hawaii	SEER 9	1975-2016	118,685	428
Iowa	SEER 9	1975-2016	314,191	1,321
New Mexico	SEER 9	1975-2016	156,830	789
Seattle-Puget Sound	SEER 9	1975-2016	418,995	1,545
Utah	SEER 9	1975-2016	161,122	591
Metropolitan Atlanta	SEER 9	1975-2016	239,639	935
Alaska	SEER 13	1992-2016	7,496	81
San Jose-Monterey	SEER 13	1992-2016	190,550	1,050
Los Angeles	SEER 13	1992-2016	719,362	5,476
Rural Georgia	SEER 13	1992-2016	13,136	51
Greater California	SEER 18	2000-2016	1,146,228	5,911
Kentucky	SEER 18	2000-2016	315,625	1,297
Louisiana	SEER 18	2000-2016	310,603	1,286
New Jersey	SEER 18	2000-2016	646,404	3,129
Greater Georgia	SEER 18	2000-2016	390,557	1,436
**TOTAL**			6,350,139	31,187

Table S3. Variables in analysis. Categorization reflects final variable composition.

Variable (SEER Variable)	Variable Name/Description per SEER	Categorization
**Exposure**		
Histology (ICD-0-3)	8490/3 (Signet Ring Cell Carcinoma)<br>814x/x (Adenocarcinoma, NOS)<br>807x/x (Squamous Cell Carcinoma, NOS)<br>824x/x (Carcinoid Tumor, Malignant)<br>816x/x (Cholangiocarcinoma) (Gallbladder/Biliary)<br>850x/x (Ductal Carcinoma) (Breast)<br>852x/x (Lobular and other Ductal Ca) (Breast)<br>812x/x, 813x/x (Transitional Cell Carcinoma, NOS & Papillary Transitional Cell Carcinoma)<br>846x/x (Papillary Serous Cystadenocarcinoma)	Signet Ring – 849x<br>Adenocarcinoma - 814x<br>Squamous Cell – 807x<br>Carcinoid – 824x<br>Cholangio. – 816x<br>Ductal – 850x<br>Lobular – 852x<br>Transition Cell – 812x,813x<br>Pap. Ser. Cys. – 846x
**Outcome**		
Mortality (vsrtsadx) (srv_time_mon)	vsrtsadx (SEER cause-specific death classification)<br>0 (Alive or dead of other cause), 1 (Dead), 8 (Dead – missing/unknown cause of death), 9 (N/A not first tumor)<br>srv_time_mon (Survival months)<br>0–9998 (in months), 9999 (Unknown)	Alive/N/A – 0,9<br>Dead – 1<br><br>Months (0–9998)
**Co-variables**		
Age (age_dx)	000-130 (Actual age of diagnosis in years), 999 (Unknown)	-
Gender (sex)	1 (Male), 2 (Female)	Male – 1<br>Female – 2
Race (race1v)	1 (white), 2 (black) 3–97 (specific races), 98 (other), 99 (unknown)	White – 1<br>Black – 2<br>Other – 3–98

Table S3. *Cont.*

Detection Stage (hst_stga (prior to 2016) & dsrpsg (2016))	hst_stga description:<br>0 (In situ — A noninvasive neoplasm; a tumor which has not penetrated the basement membrane nor extended beyond the epithelial tissue)<br>1 (Localized — An invasive neoplasm confined entirely to the organ of origin. It may include intraluminal extension where specified)<br>2 (Regional — A neoplasm that has extended 1) beyond the limits of the organ of origin directly into surrounding organs or tissues; 2) into regional lymph nodes by way of the lymphatic system; or 3) by a combination of extension and regional lymph nodes)<br>4 (Distant — A neoplasm that has spread to parts of the body remote from the primary tumor either by direct extension or by discontinuous metastasis to distant organs, issues, or via the lymphatic system to distant lymph nodes)<br>8 (Localized/Regional – Only used for Prostate cases)<br>9 (Unstaged — Information is not sufficient to assign a stage)	In situ – 0 (hst_stga) & 0x (dsrpsg)<br>Localized – 1 (hst_stga) & 1x (dsrpsg)<br>Regional – 2 (hst_stga) & 2x/3x (dsrpsg)<br>Distant – 4 (hst_stga) & 4x (dsrpsg)<br>Unknown – 9 (hst_stga) & 99/88/OC (dsrpsg)<br><br>For prostate:<br>Localized/Regional – 8 (hst_stga) & 1x/2x/3x (dsrpsg)
Grade Differentiation (grade)	1 (Grade I; grade i; grade 1; well differentiated; differentiated, NOS), 2 (Grade II; grade ii; grade 2; moderately differentiated; moderately differentiated; intermediate differentiation), 3 (Grade III; grade iii; grade 3; poorly differentiated; differentiated), 4 (Grade IV; grade iv; grade 4; undifferentiated; anaplastic), 5 (T-cell; T-precursor), 6 (B-cell; Pre-B; B-Precursor), 7 (Null cell; Non T-non B), 8 (NK cell (natural killer cell)), 9 (cell type not determined, not stated or not applicable)	Well – 1<br>Moderate – 2<br>Poor – 3<br>Undifferentiated – 4<br>Unknown – 9
Surgery (ss_surg (for cases prior to 1998) & surgprif (for cases from 1998–2016))	00 (None, no surgical procedure of primary site, diagnosed at autopsy only), 10–98 (site specific codes), 99 (unknown if surgery performed; death certificate only)	Yes – 01–98<br>No – 00
Radiotherapy (radiatnr)	0 (None/Unknown, diagnosed at autopsy), 1 (Beam radiation), 2 (Radioactive implants), 3 (Radioisotopes), 4 (Combination of 1 with 2 or 3), 5 (Radiation, NOS – method or source not specified), 6 (Other radiation – 1973-1987 cases only), 7 (Patient or patient's guardian refused radiation therapy), 8 (Radiation recommended, unknown if administered)	Yes – 1,2,4,3,4,5,6<br>No – 0,7,8
Chemotherapy (chemo_rx_rec)	0 (None/Unknown), 1 (Yes)	Yes – 1<br>No – 0

Table S4. Breakdown of SRCC cases in SEER (1975-2016), both analyzed and not analyzed.

Signet Ring Cell Cases in SEER		(1975-2016)		(1992-2016)	
**Site**	**SEER Site Recode (siterwho) ICD-O-3/WHO 2008**	**Count**	**% Total**	**Count**	**% Total**
**Sites Analyzed**		**39,695**	**94.83**	**36,810**	**94.95**
Stomach	21020 (Stomach)	23,783	56.83	21,890	56.46
Colon	21041 (Cecum), 21043, (Ascending Colon), 21044 (Hepatic Flexure), 21045 (Transverse Colon), 21046 (Splenic Flexure), 21047 (Descending Colon), 21048 (Sigmoid Colon), 21049 (Large Intestine, NOS)	6,422	15.35	6,005	15.49
Esophagus	21010 (Esophagus)	1,974	4.72	1,938	5.00
Rectum	21051 (Rectosigmoid Junction), 21052 (Rectum)	1,783	4.26	1,634	4.21
Lung	22030 (Lung and Bronchus)	1,300	3.11	1,239	3.20
Pancreas	21100 (Pancreas)	860	2.06	800	2.06
Appendix	21042 (Appendix)	829	1.98	799	2.06
Gallbladder/Biliary	21080 (Gallbladder), 21090 (Other Biliary)	689	1.65	651	1.68
Breast	26000 (Breast)	627	1.50	514	1.33
Urinary Bladder	29010 (Urinary Bladder)	533	1.27	508	1.31
Small Bowel	21030 (Small Intestine)	457	1.09	443	1.14
Ovary	27040 (Ovary)	248	0.59	218	0.56
Prostate	28010 (Prostate)	180	0.43	171	0.44
**Sites Not Analyzed**		**2,162**	**5.17**	**1,958**	**5.05**
Oral Cavity/ Pharynx	20030 (Salivary Gland), 20040 (Floor of Mouth), 20050 (Gum and Other Mouth), 20070 (Tonsil), 20090 (Hypopharynx)	11	0.03	11	0.04
Anus	21060 (Anus, Anal Canal and Anorectum)	85	0.20	81	0.20
Liver	21071 (Liver), 21072 (Intrahepatic Bile Duct)	18	0.04	18	0.05
Peritoneum	21110 (Retroperitoneum), 21120 (Peritoneum, Omentum and Mesentery)	25	0.06	23	0.06
Other Digestive	21130 (Other Digestive Organs)	523	1.25	486	1.25
Respiratory System	22010 (Nose, Nasal Cavity and Middle Ear)	10	0.03	6	0.02
Skin	25020 (Other Non-Epithelial Skin)	10	0.03	10	0.04
Female Genital System	27010 (Cervix Uteri), 27020 (Corpus Uteri), 27030 (Uterus, NOS), 27050 (Vagina), 27060 (Vulva), 27070 (Other Female Genital Organs)	82	0.18	80	0.18
Urinary System	29020 (Kidney and Renal Pelvis), 29030 Ureter), 29040 (Other Urinary Groups)	20	0.04	20	0.05
Thyroid	32010 (Thyroid)	1	0.01	1	0.01
Miscellaneous	37000 (Miscellaneous)	1,381	3.30	1,222	3.15
